# Effect of S–Se Bioisosteric Exchange on Affinity and Intrinsic Efficacy of Novel *N*-acylhydrazone Derivatives at the Adenosine A_2A_ Receptor

**DOI:** 10.3390/molecules26237364

**Published:** 2021-12-04

**Authors:** Júlia Galvez Bulhões Pedreira, Rafaela Ribeiro Silva, François G. Noël, Eliezer J. Barreiro

**Affiliations:** 1 Laboratory of Evaluation and Synthesis of Bioactive Substances (LASSBio), Institute of Biomedical Sciences, Federal University of Rio de Janeiro (UFRJ), Rio de Janeiro 21944-971, Brazil; jugalvez@gmail.com; 2 Graduate Program of Chemistry (PGQu), Chemistry Institute, Federal University of Rio de Janeiro (UFRJ), Rio de Janeiro 21941-909, Brazil; 3 Laboratory of Biochemical and Molecular Pharmacology, Institute of Biomedical Sciences, Federal University of Rio de Janeiro (UFRJ), Rio de Janeiro 21944-971, Brazil; rafaela_ribe@yahoo.com.br (R.R.S.); fgagnoel@gmail.com (F.G.N.); 4 Nacional Institute of Science & Technology in Drugs and Medicines (INCT-INOFAR), Federal University of Rio de Janeiro (UFRJ), Rio de Janeiro 21944-971, Brazil

**Keywords:** *N*-acylhydrazone derivatives, A_2A_ receptor, sulfur-selenium isosterism, conformational effect, chalcogen interaction, *N*-methylation effect

## Abstract

In this work, we evaluated the conformational effect promoted by the isosteric exchange of sulfur by selenium in the heteroaromatic ring of new *N*-acylhydrazone (NAH) derivatives (**3**–**8**, **13**, **14**), analogues of the cardioactive compounds LASSBio-294 (**1**) and LASSBio-785 (**2**). NMR spectra analysis demonstrated a chemical shift variation of the iminic C*sp*^2^ of NAH S/Se-isosters, suggesting a stronger intramolecular chalcogen interaction for Se-derivatives. To investigate the pharmacological profile of these compounds at the adenosine A_2A_ receptor (A_2A_R), we performed a previously validated functional binding assay. As expected for bioisosteres, the isosteric-S/Se replacement affected neither the affinity nor the intrinsic efficacy of our NAH derivatives (**1**–**8**). However, the *N*-methylated compounds (**2**, **6**–**8**) presented a weak partial agonist profile at A_2A_R, contrary to the non-methylated counterparts (**1**, **3**–**5**), which appeared as weak inverse agonists. Additionally, retroisosterism between aromatic rings of NAH on S/Se-isosters mimicked the effect of the *N*-methylation on intrinsic efficacy at A_2A_R, while *meta*-substitution in the phenyl ring of the acyl moiety did not. This study showed that the conformational effect of NAH-*N*-methylation and aromatic rings retroisosterism changed the intrinsic efficacy on A_2A_R, indicating the S/Se-chalcogen effect to drive the conformational behavior of this series of NAH.

## 1. Introduction

**Figure 1:**
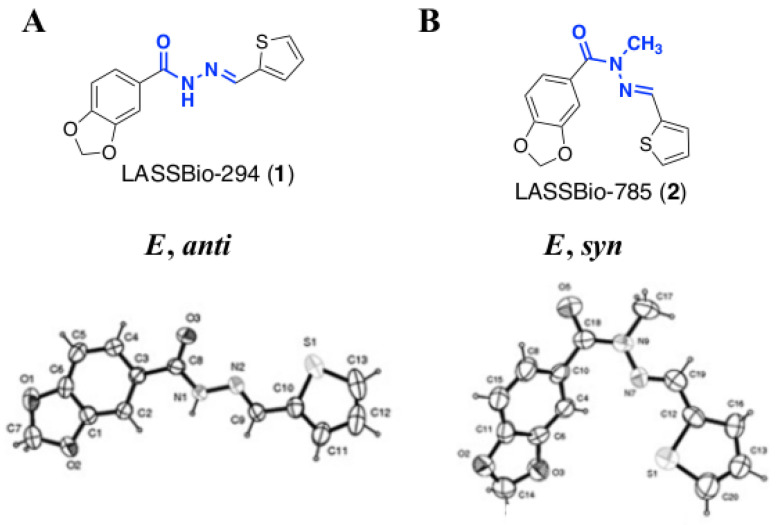
Conformational effect by *N*-methylation of the amide of NAH function, from *anti-* to *syn-* conformation of cardioactive compounds LASSBio-294 (**1**) and LASSBio-785 (**2**). (**A**) Chemical and X-ray structure of LASSBio-294 (**1**); (**B**) Chemical and X-ray structure of LASSBio-785 (**2**).

One of the main challenges in medicinal chemistry is the identification of the bioactive conformation of small molecules. During the development of bioactive *N*-acylhydrazones (NAH) derivatives [1,2], a significant difference between two homologues compounds, LASSBio-294 (**1**) and LASSBio-785 (**2**) [3,4,5,6] (Figure 1), was identified. Indeed, pharmacological studies showed that these similar compounds have distinct cardiovascular effects. LASSBio-294 (**1**) was more inotropic than vasodilatory, contrary to LASSBio-785 (**2**), a more potent vasodilator without a cardiac effect [7,8,9,10,11,12].

Preliminary data obtained from CEREP (Study Number: 15180, 2008) on a panel of GPCRs indicated that both LASSBio-294 (**1**) [13] and LASSBio-785 (**2**) (unpublished data) had a moderate affinity for the adenosine A_2A_ receptor (A_2A_R), a putative target for these drugs.

**Figure 2:**
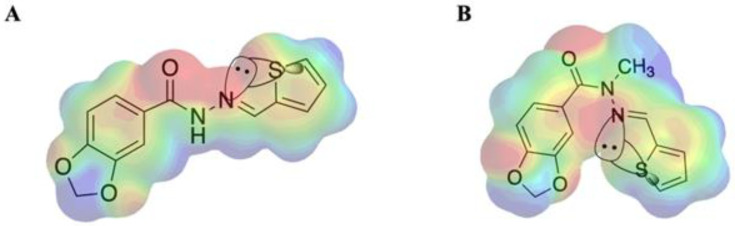
Representation of chalcogen interaction between the electron pair of the iminic nitrogen and the antibonding orbital in the thiophene ring. (**A**) Electrostatic potential map of LASSBio-294 (**1**); (**B**) Electrostatic potential map of LASSBio-785 (**2**). Red shadow represents the negatively charged area; blue shadow represents the positively charged area.

The careful structure–property–activity relationship of LASSBio-294 (**1**) and LASSBio-785 (**2**) demonstrated that the *N*-methylation of the NAH moiety has a profound effect on this scaffold [14], leading to different electronical profiles (identified by ^1^H and ^13^C NMR studies) and physicochemical properties [15,16]. Follow up studies by X-ray diffraction indicated that the two compounds adopted an unexpected distinct conformation [15], with **1** having a hair-pin like conformation (Figure 1A) and **2** a U-shape conformation (Figure 1B) [17]. In both conformations (Figure 1A,B), it is outstanding to note that the heteroatom of the ring at the imine double bond adopted the same steric orientation viz-à-viz the N*sp^2^* corresponding to a typical chalcogen interaction. This conformational behavior was investigated, confirming the steric orientation of the 2-thiophene ring at the terminus of LASSBio-294 (**1**) and LASSBio-785 (**2**), as an accountable σ-hole or chalcogen interaction [18,19,20] between *S*-atom and the electron-pair of the N-*sp^2^* atom of the NAH moiety [21]. This observation suggests that this kind of interaction could be helpful in drug design to address conformational restrictions in bioactive NAH derivatives [21] (Figure 2).

Since selenium, an isoster of sulfur in the chalcogen family, has a comparably higher polarizability, we hypothesized that a classical isosteric exchange of S to Se [22,23] in the five-member ring could intensify the chalcogen interaction. Considering this, we prepared a series of new NAH compounds structurally related to LASSBio-294 (**1**) and LASSBio-785 (**2**). The aim was to evaluate how the isosteric change, combined with the new chalcogen-induced conformation, would affect the affinity and/or intrinsic efficacy of these compounds at the adenosine A_2A_R [13,24].

Our results showed that *N*-methylation of Se-containing NAH compounds, changed their intrinsic efficacy, with the *N*-methylated derivatives (**2**, **6**, **7**, and **8**) having a weak partial agonist profile in contrast to the non-methylated counterparts (**1**, **3**, **4**, and **5**), which appeared as weak inverse agonists. It was also observed that isosteric S–Se replacement at the five member ring did not affect affinity or intrinsic efficacy at A_2A_R. Moreover, we observed that isosteric change has a marked conformational effect in the selenophenic derivatives (**3**, **5**, **6**, **8**, and **12**) probably due the stronger Se-N*sp^2^* intramolecular chalcogen interaction, which could be able to mimic the *N*-methylation effect observed in the S-isostere LASSBio-785 (**2**).

## 2. Results and Discussion

### 2.1. Molecular Design

**Figure 3:**
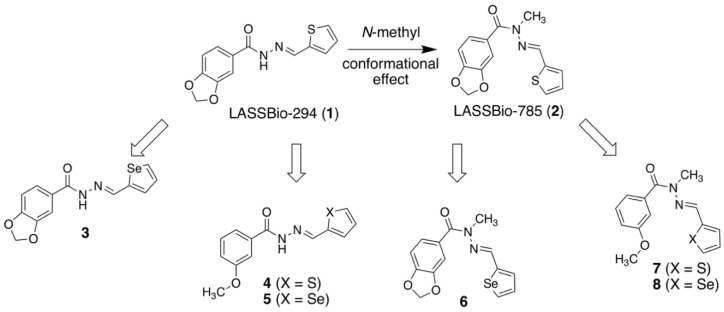
Molecular design of novel *N*-acylhydrazone derivatives. Series of isosteric NAH analogues to the previously described LASSBio-294 (**1**) and LASSBio-785 (**2**).

The initial approach was to synthesize selenium isosters of **1** and **2**, the new NAH derivatives **3** and **6** (Figure 3). Additionally, previous studies of structurally similar compounds to **1** and **2** demonstrated that the *meta*-position with a methoxy group in the phenyl ring leads to increased potency in the adenosine receptors [25]. Hence, we also prepared novel isosteric analogues of *meta*-substituted compounds (**4**, **5**, **7**, and, **8**, Figure 3).

### 2.2. Isosteric Derivatives (***3**–**8***)

#### 2.2.1. Chemistry

**Scheme 1:**
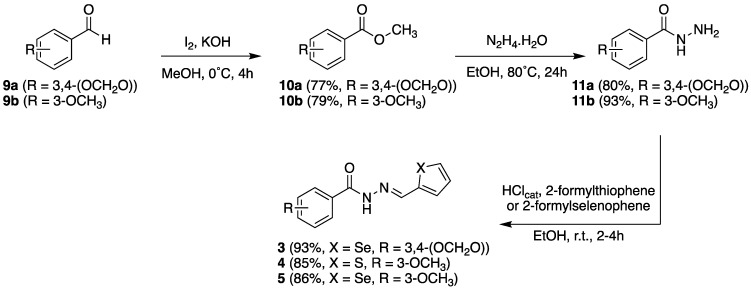
Synthetic scheme for preparation of *N*-acylhydrazonic compounds **3**, **4,** and **5**. Reaction conditions and yields leading to NAH compounds **3**–**5**.

The synthesis of the new NAH compounds (**3**–**5**) was performed in three steps [4]. First, the esters (**10a** and **10b**) [26,27] were prepared by Yamada oxidation in methanol of the corresponding commercial aldehydes (**9a** and **9b**) [28], and then submitted to hydrazinolysis reaction in ethanol to produce the key hydrazide intermediates **11a** and **11b** (Scheme 1) [29,30]. Finally, the desired NAH derivatives (**3**–**5**) were obtained in high overall yields by an acid-catalyzed condensation reaction with the corresponding aldehydes (2-formylthiophene or 2-formylselenophene, Scheme 1).

**Scheme 2:**
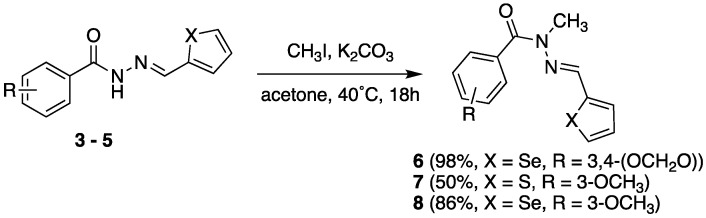
Synthetic scheme for methylated compounds **7**–**8**. Reaction conditions and yields for the methylation of NAH compounds **3**–**5** to afford compounds **6**–**8**.

Next, the *N*-methylation reaction was performed using iodomethane and potassium carbonate in acetone, at mild heating [15] (Scheme 2). The *N*-CH_3_-NAH compounds **6**–**8** were obtained after chromatographic column purification.

Detailed ^1^H-NMR analysis of the isosteric pairs (**1** and **3**, **4** and **5**, **2** and **6**, **7** and **8**) demonstrated the electronic effect of the chalcogen modification (Table 1). All signals related to hydrogens of the heterocycle ring have a downfield effect upon substitution of sulfur to selenium, suggesting that its polarizability further deshields the neighboring hydrogens. No shield effect was observed on the -N=C**H**-iminic proton (Table 1) of the NAH derivatives (**1**–**8**), with all signals within the range of δ 8.5–8.6 ppm for NAH derivatives **1**, **3**–**5** and δ 8.1–8.2 ppm for *N*-methylated compounds **2**, **6**–**8**. These unchanged shifts are, however, a strong indication that the *E* isomerism of the iminic bond was not affected in the new structures [31].

For the ^13^C-NMR spectra, a significant difference was observed in the shift of the iminic carbon atom between the isosters pairs **1** and **3**; **4** and **5** (Supplementary Material, Table S1). Displacements in the shifts of carbons can also be correlated to the intramolecular chalcogen interaction [32,33]. A shift of almost 5 ppm was observed between the isosteric pairs (e.g., compounds **1** and **3** (139.2 ppm to 143.9 pm)). This indicates a weakening of the resonance system between imine and the selenophene ring as a consequence of the σ-hole interaction between Se-N*sp^2^* in isosteric compounds **3**, **5**, **6**, and **8**. All of this suggests a stronger intramolecular interaction on the selenophenic derivatives (**3**, **5**, **6**, and **8**).

#### 2.2.2. Pharmacological Evaluation

The ability of our compounds to interact with the A_2A_ receptor (A_2A_R) was evaluated through competition for the binding of [^3^H]-ZM241385 used as a probe for the A_2A_R present endogenously in rat striatum [24].

In the basal conditions (medium I, Table 2), the isosteric pairs **1** and **3**, **4** and **5**, **2** and **6**, and **7** and **8** had similar IC_50_ values (Table 2). Therefore, the isosteric replacement of S by Se did not affect the affinity of our compounds. NAH *N*-methylation also has no significant effect on affinity for A_2A_R, albeit there is a tendency for a small decrease in affinity. This result indicates that the change of conformation due to the *N*-methylation has no significant effect on the binding affinity of these ligands, at least for the A_2A_R in its high-affinity conformation (medium I, Table 2).

**Figure 4:**
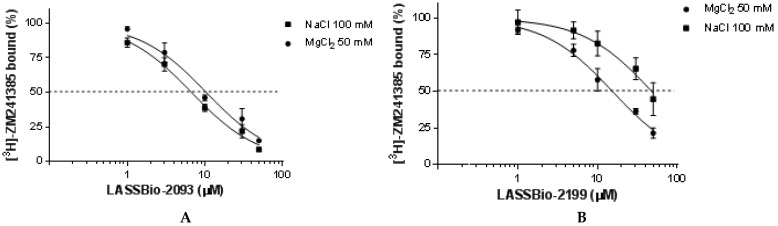
Na^+^-shift assay for the estimation of the intrinsic efficacy of the NAH derivatives **5** and **8** at the A_2A_ receptor (A_2A_R) in rat striatum membrane preparation. Competition curves were performed using the antagonist radioligand (0.5 nM [^3^H]-ZM241385) in the presence of 50 mM MgCl_2_ or 100 mM NaCl. Each curve represents the averaged curve (±S.E.) from three independent paired experiments (in triplicate). (**A**) LASSBio-2093 (**5**); (**B**) *N*-methylated LASSBio-2199 (**8**).

As the pharmacodynamic properties of a drug depend not only on its affinity for a receptor but also on its capacity to activate or block the receptor (i.e., efficacy), we also used the Na+-shift assay previously validated for quantifying the intrinsic efficacy of our compounds [24]. This functional binding assay is based on the extended ternary complex model of G-protein coupled receptor (GPCR). According to this model, the activated conformation of GPCR is the one coupled to a G-protein. Moreover, the affinity of an agonist is much higher for the receptor in this conformation than for the uncoupled receptor [34]. In our assay, the substitution of Mg2+ (a divalent cation that favors the formation of the ternary complex) by Na+ (a negative allosteric modulator of agonist binding and activation in many GPCRs) shifts the competition curve of agonists, but not antagonists, to the right [24,35]. On the other hand, a shift of the curve to the left indicates that the compound is an inverse agonist. This profile was observed with all four non-methylated *N*-acylhydrazone derivatives (compounds **1** and **3**–**5**), as exemplified in Figure 4A for LASSBio-2093 (**5**), and shown in Figure S1A,C,E for the other derivatives. Note that Na+-shifts to the left observed with these four compounds were small, but statistically significant, as indicated by a ratio (II/I) lower than one (*p* < 0.05, Table 2). 

On the other hand, the competition curves with the *N*-methyl-*N*-acylhydrazone compounds (**2**, **6**–**8**) shifted to the right in the presence of Na^+^, as shown in Figure 4B for LASSBio-2199 (**8**), and in Figure S1B,D,F for the other derivatives. The small but statistically significant (*p* < 0.05, Table 2) shift to the right (II/I ratio) indicates that these compounds act as weak partial agonists in our conditions. Note that this assay was validated previously [24] by successful discrimination between classical A_2A_R full agonists (CGS21680, NECA, and adenosine, with IC_50_ ratios = 13–14) and partial agonists (LUF5834 and regadenoson, with IC_50_ ratios equal to 3 and 10, respectively).

Since the IC_50_ ratios (II/I) were similar for the isosteric pairs (**1** and **3**, **4** and **5**, **2** and **6**, and **7** and **8**), we can conclude that isosteric replacement of S by Se did not affect the intrinsic efficacy of compounds, probably because the conformational behavior of both is similar. On the other hand, *N*-methylation of the *N*-acylhydrazone derivatives changed the intrinsic efficacy while simple *meta*-substitution in the phenyl ring did not. These results can be explained by the conformational change induced by NAH methylation as previously observed for LASSBio-785 (**2**) [15,17].

### 2.3. Retroisosteric Derivatives (***13*** and ***14***)

**Figure 5:**
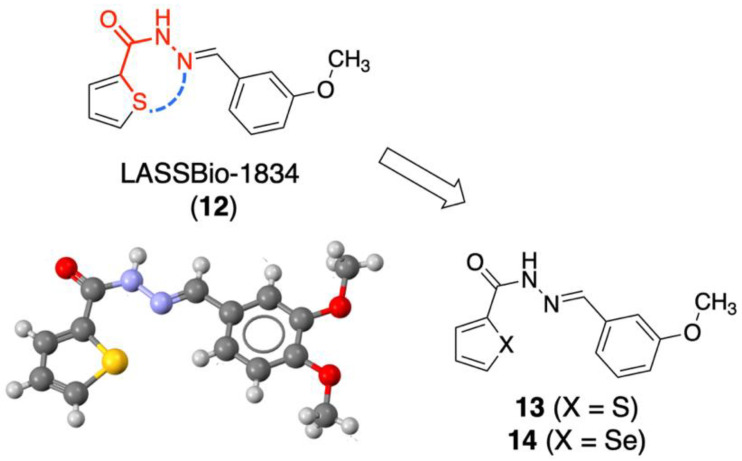
Chemical structure of LASSBio-1834 (**12**) and new isosteric analogues **13** and **14**. Representation of the *syn*-periplanar conformation of the amide bond in the NAH due to chalcogen interaction, as observed through X-ray analysis.

Previous results from our working group [36] on NAH derivatives with the thiophene ring on the acyl-terminus have demonstrated that the molecule displays an important intramolecular 1–5 σ-hole-S-N*sp^2^* interaction. The geometry of such interaction provides a better orientation for the overlap between the non-bonded electronic pair of the nitrogen and the σ-hole in the heterocycle. This kind of interaction in NAH systems leads to a conformational modification in the amide bond to *syn*-periplanar, as observed for *N*-CH_3_-NAH derivatives. The X-ray diffraction analysis of compound LASSBio-1834 (**12**, Figure 5) confirms this assumption [36].

In this context, we decided to investigate how the herein previously described structural and electronic effects promoted by the bioisosteric change between S and Se would apply to the retroisosteric analogs (**13** and **14**). These retroisosters were designed by the interchange between the five-ring heterocycle and the oxygenated phenyl ring, substituents of acyl and imine terminus, respectively. Due to the higher activity of 3-methoxy substituted compounds **4** and **5** in the previous assays (as demonstrated in Table 2), this structural pattern of the phenyl ring in the new retroisosteric derivatives (**13** and **14**) was preserved.

#### 2.3.1. Chemistry

**Scheme 3:**
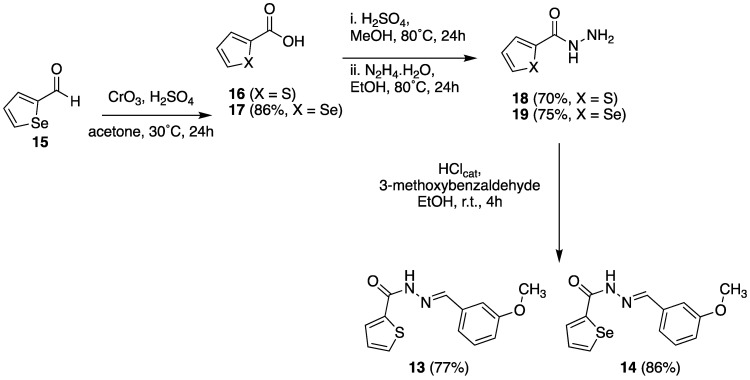
Synthetic scheme for the preparation of retroisosteric derivatives **13** and **14**. Reaction conditions and yields for the synthesis of thiophenic and seleonphenic hydrazides and subsequent condensation to obtain retroisosteric NAH **13** and **14**.

For the preparation of the thiophenic hydrazide (**18**) [37] intermediate (Scheme 3), initially the thiophene-2-carboxylic acid (**16**) was converted to methyl ester (**20**, synthetic description in Supplementary Materials) by standard acid-catalyzed Fischer esterification conditions [38]. The hydrazinolisis reaction leads to intermediate **18**, followed by acid-catalyzed condensation with the 3-methoxybenzaldehyde [4] to afford compound **13** (Scheme 3). The selenophenic hydrazide intermediate **19** [39] (Scheme 3) was obtained first by Jones oxidation [40] of 2-formylselenophene (**15**) to afford the carboxylic acid (**17**, Supplementary Materials). It was then converted into the methyl ester **21** (Supplementary Materials) by Fischer conditions in methanol, followed by hydrazinolysis to afford **19**. Again, acid-catalyzed condensation of selenohydrazide (**19**) with 3-methoxybenzaldehyde was performed in standard conditions, previously described, affording the desired new NAH derivative **14** in appropriated yield (Scheme 3).

**Figure 6:**
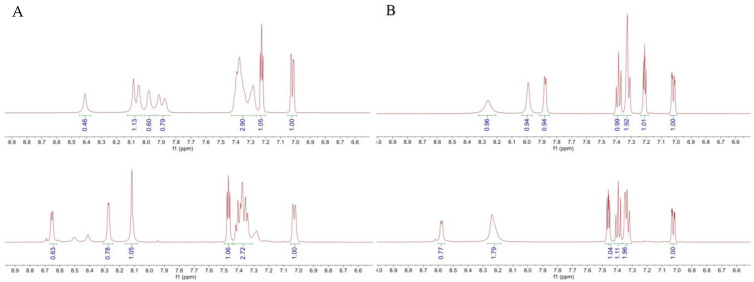
Comparison between spectra of compounds **11** and **12** at different temperatures and coalescence of doubled signals. (**A**) ^1^H NMR spectra of **11** (top) and **12** (bottom) at 25 °C; (**B**) ^1^H NMR of **11** (top) and **12** (bottom) at 80 °C.

The retroisosterism approach [23] used in **13** and **14** tends to favor a distinct chalcogen intramolecular interaction, which changes the conformational behavior of the amide bond. Since the *syn*-periplanar conformation is favored by the σ-hole 1,5-N*sp^2^*^…^S, and 1,5-N*sp^2^*^…^Se interaction, these new retrosisosteric NAH derivatives **13** and **14** could possibly adopt a similar conformational behavior to *N*-methylated analogs **7** and **8**. In fact, it was observed in the ^1^H NMR that there is a duplicity of signals related to their conformation (Figure 6A). This was confirmed by coalescence of the related signals with the higher temperature experiment (Figure 6B). This indicates the possibility of the chalcogen interaction like LASSBio-1834 (**10**) in the new NAH compounds **13** and **14**.

#### 2.3.2. Pharmacological Evaluation

As for the first set of compounds, we performed the Na^+^-shift assay with the retroisosteric analogues LASSBio-2278 (**13**) and LASSBio-2279 (**14**) to evaluate their pharmacological profile (Figure S2). Comparing the retroisosteres **13** and **14** with their counterparts (**4** and **5**), the main difference relates to their intrinsic efficacy. Indeed, the IC_50_ ratio (II/I) was higher than one (Table 3), indicating that compounds **13** and **14** are weak partial agonists. Similarly, to NAH *N*-methylation, simple retroisosterism changed the intrinsic efficacy of our compounds on A_2A_R (Table 3).

## 3. Materials and Methods

All reagents and (anhydrous) solvents are commercially available and were used without further purification. NMR spectra were obtained at UFRJ with a VARIAN 400-MR and 500-MR (IPPN-UFRJ) (Varian Inc., Palo Alto, CA, USA). The spectra were obtained in the indicated solvent and calibrated against the residual proton peak of the deuterated solvent. Chemical shifts (δ) are reported in parts per million. Mass spectra were obtained on an Esquire 6000-ESI Ion Trap MSn System Bruker Daltonics (LASSBio-UFRJ) (Bruker, Billerica, MA, USA).

4-(−2-[7-amino-2-{2-furyl}{1,2,4}triazolo{2,3 a}{1,3,5} tria-zin-5-ylamino]ethyl) phenol (ZM241385), 5′-(*N*-ethylcarboxamido) adenosine (NECA), 1,4-bis-[2-(5-phenyloxazolyl)]-benzene (POPOP) and 2,5-diphenyloxazole (POP) and other reagents were purchased from Sigma-Aldrich Co nowadays Merck chemicals, and [^3^H]-ZM241385 (50 Ci/mmol) from American Radiolabeled Chemicals, Inc., Maryland Heights, MI, USA. Stock solutions of our compounds were prepared in DMSO and at the final concentration used (less than 0.5%), DMSO had no effect on our biological assays.

### 3.1. Synthesis of N-Acylhydrazones (***3**–**8***, ***13***, and ***14***)

The corresponding hydrazide (**11a, 11b, 18** or **19**) [29,30,37,39] was solubilized in EtOH solution with 1% *v*/*v* HCl (0.2–0.3 M). The aldehsyde was added, and the reaction stirred at room temperature. After ca. 30 min to 1 h, a precipitate was formed. The reaction was monitoressd via TLC (20–70% EtOAc/*n*-hex) until completion. The work up was made by evaporating the solvent on a rotary evaporator, the addition of water, and filtration under vacuum on a Büchner funnel.

#### 3.1.1. *N*’-(Selenophen-2-yl-methylene)benzo[d][1,3] dioxole-5-carbohydrazide (**3**)

Compound **3** was prepared according to the procedure described in Section 3.1 to afford a yellow solid (0.24 g 0.76 mmol) with 93% yield; m.p. 215 °C. ^1^H NMR (DMSO-*d^6^*, 400 MHz) δ 11.68 (1H, s, OCNH); 8.60 (1H, s, N=CH); 8.23 (1H, d, *J* = 5.4 Hz, H5’); 7.62 (1H, d, *J* = 3.3 Hz, H3’); 7.49 (1H, d, *J* = 8.0 Hz, H6); 7.42 (1H, s, H2,); 7.35 (1H, dd, *J_1_* = 3.3 Hz, *J_2_* = 5.4 Hz, H4’); 7.05 (1H, d, *J* = 8.0 Hz, H5,) 6.13 (2H, s, O-CH_2_-O). ^13^C NMR (DMSO-*d^6^*, 100 MHz) δ 162.6 (OCNH); 150.7 (C4); 147.9 (C3); 145.5 (C2’); 145.2 (N=CH-); 134.4 (C5’); 133.7 (C3’); 133.6 (C1); 130.8 (C4′); 123.2 (C6); 108.6 (C5); 108.0 (C2); 101.9 (O–CH_2_O). HRMS(ESI) m/z: [M + Na]^+^ Calcd. for C_13_H_10_N_2_O_3_SeNa 344.9754; Found 344.9749.

#### 3.1.2. 3-Methoxy-*N’*-(thiophen-2-yl-methylene)benzohydrazide (**4**)

Compound **4** was prepared according the procedure described in Section 3.1 to afford a white solid (0.23 g, 0.76 mmol) with 85% yield; m.p. 167 °C. HRMS(ESI) m/z: [M + Na]^+^ Calcd. for C_13_H_12_N_2_O_2_SNa 283.0517; Found 283.0511. This compound has been previously described in the literature [41].

#### 3.1.3. 3-Methoxy-*N’*-(selenophen-2-yl-methylene)benzohydrazide (**5**)

Compound **5** was prepared according to the procedure described in Section 3.1 to afford a yellow solid (0.23 g, 0.76 mmol) with 86% yield; m.p. 174 °C. ^1^H NMR (DMSO-*d^6^*, 400 MHz) δ 11.85 (1H, s, OCNH), 8.66 (1H, s, N=CH), 8.24 (1H, d, *J* = 5.4 Hz, H5′), 7.62 (1H, d, *J* = 3.4 Hz, H3′), 7.47 (1H, d, *J* = 7.5 Hz, H2), 7.45–7.42 (2H, m, H4 and H6), 7.36 (1H, t, *J* = 4.5 Hz, H4′), 7.15 (1H, d, *J* = 9 Hz, H5), 3.83 (3H, s, OCH_3_). ^13^C NMR (DMSO-*d^6^*, 100 MHz) δ 162.7 (OCNH), 159.3 (C3), 145.1 (C2′), 144.6 (N=CH), 133.9 (C5′), 133.1 (C3′), 130.1 (C6 and C4′), 129.5 (C2), 119.8 (C1), 117.2 (C4), 112.6 (C5). HRMS(ESI) m/z: [M + Na]^+^ Calcd. for C_13_H_12_N_2_O_2_SeNa 330.9962; Found 330.9956.

#### 3.1.4. *N’*-(3-Methoxybenzylidene)thiophene-2-carbo-hydrazide (**13**)

Compound **13** was prepared according to the procedure described in Section 3.1 to afford a yellow solid (0.21 g, 0.8 mmol) with 77% yield; m.p. 200 °C. ^1^H NMR (DMSO-*d^6^*, 500 MHz) δ 11.88 (1H, s, OCNH), 8.40 (1H, s, N=CH), 8.08–8.05 (1H, m, H5), 7.99–7.88 (1H, s, H3), 7.42–7.28 (3H, m, H2′, H4′ and H6′), 7.22 (1H, d, *J_1_* = 4.3 Hz, *J_2_* = 5.4 Hz, H4′), 7.01 (1H, d, *J* = 7.9 Hz, H5′), 3.83 (3H, s, OCH_3_); ^13^C NMR (DMSO-*d^6^*, 125 MHz) δ 161.4 (OCNH), 159.6 (C2′), 147.6 (N=CH), 143.9 (C5), 136.6 (C3), 135.3 (C3′), 135.0 (C2), 130.2 (C6′), 129.8 (C4′), 126.7 (C4), 120.0 (C2′), 116.0 (C5′), 111.8 (C1′), 55.3 (OCH_3_). HRMS(ESI) m/z: [M + Na]^+^ Calcd. for C_13_H_12_N_2_O_2_SNa 283.0517; Found 283.0511. This compound has been previously described in the literature [42].

#### 3.1.5. *N’*-(3-Methoxybenzylidene)selenophene-2-ca-rbohydrazide (**14**)

Compound **14** was prepared according to the procedure described in Section 3.1 to afford a yellow solid (70 mg, 0.2 mmol) with 86% yield; m.p. 151 °C. ^1^H NMR (DMSO-*d^6^*, 500 MHz) δ 11.85 (1H, s, OCNH), 8.65 (1H, d, *J* = 5.4 Hz, H5), 8.27 (1H, d, *J* = 3.0 Hz, H3), 8.12 (1H, s, N=CH), 7.47 (1H, t, *J* = 4.7 Hz, H4), 7.42–7.34 (3H, m, H2′, H4′ and H6′), 7.02 (1H, d, *J* = 7.0 Hz, H5′), 3.83 (3H, s, OCH_3_). ^13^C NMR (DMSO-*d^6^*, 125 MHz) δ 162.3 (OCNH), 159.6 (C2′), 144.1 (N=CH), 142.7 (C5), 137.1 (C3), 135.4 (C3′), 135.3 (C2), 130.2 (C6′), 128.8 (C4), 120.2 (C4′), 116.2 (C5′), 111.8 (C1), 55.23 (OCH_3_). HRMS(ESI) m/z: [M + Na]^+^ Calcd. for C_13_H_12_N_2_O_2_SeNa 330.9962, found 330.9956.

### 3.2. N-Methylation of N-Acylhydrazones (***6***, ***7*** and ***8***)

The corresponding *N*-acylhydrazone (**3**, **4**, or **5**) was dissolved in acetone (0.1–0.2 M) in a round-bottom flask, and K_2_CO_3_ (3 eq.) and CH_3_I (4 eq.) were added to the reaction mixture. The flask was attached to a reflux condenser and the reaction stirred and heated to 40 °C. TLC control (20 or 50% of EtOAC/*n*-hex) was made at the completion of the reaction. Quenching was conducted by evaporating the solvent on a rotary evaporator and the addition of water. The product was either filtrated under vacuum or extracted with EtOAc (3 × 15 mL), dried over Na_2_SO_4_, and the solvent evaporated. Further purification was carried out by column chromatography (5–50% EtOAc/*n*-hex) and recrystallization in EtOH followed by filtration under vacuum, if possible.

#### 3.2.1. *N*-Methyl-*N*’-(selenophen-2-yl-methylene) benzo[d][1,3]dioxole-5-carbohydrazide (**6**)

Compound **6** was prepared according to the procedure described in Section 3.2 to afford a yellow solid (0.1 g, 0.3 mmol) with 98% yield; m.p. 125 °C; ^1^H NMR (DMSO-*d^6^*, 400 MHz) δ 8.16 (s, 1H, N=CH), 8.10 (d, *J* = 5.5 Hz, 1H, H5’), 7.56 (d, *J* = 3.7 Hz, 1H, H3’), 7.31 (dd, *J* = 5.4, 3.9 Hz, 1H, H4’), 7.21 (d, *J* = 8.1 Hz, 1H, H2), 7.18 (s, H6), 6.96 (d, *J* = 8.1 Hz, 1H, H5), 6.08 (s, 1H, -OCH_2_O-), 3.44 (s, 3H, O=CNCH_3_); ^13^C NMR (DMSO-*d^6^*, 100 MHz) δ 162.1 (OCNCH_3_), 157.89, 149.3 (C2’), 146.7 (C4), 146.7 (C4), 137.1 (N=CH), 133.5 (C5’), 132.5 (C3’), 131.0 (C4’), 128.8 (C1), 125.1 (C6), 111.1 (C2), 106. 8 (C5), 101.6, (-OCH_2_O-) 29.8 (CH_3_). HRMS(ESI) m/z: [M + Na]^+^ Calcd. for C_14_H_12_N_2_O_3_SeNa 358,9911; Found 358.9905.

#### 3.2.2. 3-Methoxy-*N*-methyl-*N*’-(thiophen-2-yl-methylene)benzohydrazide (**7**)

Compound **7** was prepared according to the procedure described in Section 3.2 to afford a brown oil (52 mg, 0.19 mmol) with 50% yield. ^1^H NMR (DMSO-*d^6^*, 400 MHz) δ 8.24 (s, 1H, N=CH), 7.52 (d, *J* = 5 Hz, 1H, H5’), 7.38 (d, *J* = 3.50 Hz, 1H, H3’), 7.35 (t, *J* = 8 Hz, 1H, H5), 7.18 (m, 2H, H2 e H6), 7.09 (dd, *J* = 5.1, 3.5 Hz, 1H, H4’), 7.05 (dd, J = 3 Hz, 8 Hz, 1H, H4), 3.79 (s, 3H, H_3_CO-Ar), 3.46 (s, 3H, O=CNCH_3_). ^13^C NMR (DMSO-*d^6^*, 100 MHz) δ 169.1 (OCNH), 158.2 (C3), 138.0 (N=CH), 136.4 (C2), 135.9 (C1), 130.1 (C5′), 128.4 (C3′), 128.2 (C4′), 127.8 (C2), 121.9 (C6), 116.1 (C4), 114.6 (C5), 55.2 (OCH_3_), 29.0 (NCH_3_). HRMS(ESI) m/z: [M + Na]^+^ Calcd. for C_13_H_12_N_2_O_2_SNa 297.0674; Found 297.0667.

#### 3.2.3. 3-Methoxy-*N*-methyl-*N’*-(thiophen-2-yl-methylene)benzohydrazide (**8**)

Compound **8** was prepared according to the procedure described in Section 3.2 to afford a brown oil (89 mg, 0.27 mmol) with 86% yield. ^1^H NMR (DMSO-*d^6^*, 400 MHz) δ 8.20 (s, 1H, N=CH), 8.09 (d, 1H, *J* = 5.1 Hz, H5’), 7.56 (d, 1H, *J* = 3.50 Hz, H3’), 7.35 (t, 1H, *J* = 8.2 Hz, H5), 7.30 (dd, 1H, *J* = 5.1, 3.5 Hz, H4’), 7.13 (m, 2H, H2 e H6), 7.05 (dd, 1H, J = 8.2, H4), 3.79 (s, 3H, H_3_CO-Ar), 3.46 (s, 3H, OCNCH_3_). ^13^C NMR (DMSO-*d^6^*, 100 MHz) δ 168 (OCNH), 158.2 (C3), 146.0 (N=CH), 137.6 (C2), 136.4 (C1), 133.3 (C5′), 132.2 (C3′), 130.2 (C4′), 128.4 (C2), 121.8 (C6), 116.1 (C4), 114.5 (C5), 55.2 (OCH_3_), 30.7 (NCH_3_). HRMS(ESI) m/z: [M + Na]^+^ Calcd. for C_13_H_12_N_2_O_2_SeNa 345.0118; Found 345.0121.

### 3.3. Biological Assays

Radioligand binding assays evaluating the ability of our compounds to interact with the A_2A_ receptor (A_2A_R) were performed as previously described [24]. Adult male Wistar rats (200–300 g) were euthanized by decapitation, their brains were immediately removed on ice, and the striatum dissected and stored in liquid nitrogen until use. After thawing, the tissues were homogenized in a Potter apparatus with a motor-driven Teflon pestle at 4 °C in 20 volumes per gram of tissue of ice-cold Tris–HCl 50 mM buffer (pH 7.4) containing MgCl_2_ 8 mM and EDTA 5 mM. The resulting suspension was ultracentrifuged at 48.000 g_max_ at 4 °C for 20 min. The pellet was resuspended in the same buffer and incubated at 37 °C for 10 min for endogenous neurotransmitter removal. This suspension was cooled on ice and ultracentrifuged twice at 48.000 g_av_ for 10 min at 4 °C. The final pellet was resuspended in buffer yielding 1.5 mL/g tissue and stored in liquid nitrogen until use. The protein concentration was determined by the method of Lowry et al. [43] using bovine serum albumin as the standard.

Rat striatum membrane preparation (150 µg protein) and 0.5 nM [^3^H]-ZM241385 were incubated at 25 °C for 60 min under yellow light in a buffer solution of Tris–HCl 50 mM (pH 7.4) containing EDTA 1 mM and MgCl_2_ 50 mM or 100 mM NaCl, in the absence or presence of the compounds. The non-specific binding was estimated using 30 µM NECA.

After incubation, samples were rapidly diluted with 3 × 4 mL Tris–HCl 5 mM (pH 7.4) and immediately filtered under vacuum on glass fiber filters (GMF 3; Filtrak, Germany) previously soaked in 0.5% polyethyleneimine. Filters were then dried, immersed in a scintillation mixture (POPOP 0.1 g/L and POP 4.0 g/L in toluene), and the radioactivity retained in the filters was counted using a Packard Tri-Carb 1600 TR liquid scintillation analyzer (Perkin Elmer Waltham, Massachusetts, USA.) 

In order to determine the profile of intrinsic efficacy of our compounds at the A_2A_R, we used the Na^+^-shift assay that we previously validated [24]. Paired competition curves for the binding of [^3^H]-ZM241385 were performed either in the presence of 50 mM MgCl_2_ or 100 mM NaCl. The concentration–response curves were analyzed by non-linear regression using the model of “one site competition” for determination of the median inhibitory concentration (IC_50_). The Na^+^-shift was calculated by dividing the IC_50_ value obtained in the medium containing NaCl by the IC_50_ value obtained in the medium containing MgCl_2_ and statistically analyzed by a paired t test performed on the log IC_50_ values (GraphPad Prism 6.0, GraphPad Software, Inc., San Diego, CA, USA), as previously described [24].

## 4. Conclusions

In this report, we explored the isosteric relationship between sulfur and selenium and its implication on the intramolecular interaction between the iminic nitrogen and the antibonding orbital of the C–X bond on heterocyclic residues of *N*-acylhydrazone (NAH) scaffolds. We showed that the higher polarizability of selenium has a deshielding effect on the neighboring hydrogens and seems to influence their electronical profile. However, this isosteric exchange did not modify the affinity or intrinsic efficacy of the compounds at the A_2A_ receptor (A_2A_R). On the other hand, NAH *N*-methylation significantly inverted the intrinsic efficacy of these compounds (from weak inverse agonists to partial agonists).

Present results obtained from rational simple structural modification in these series of *N*-acylhydrazone derivatives illustrated how the ligand conformation has an influence on molecular recognition by the bioreceptor.

## Figures and Tables

**Table 1:** ^1^H-NMR (400 MHz, DMSO-*d_6_*, 25 °C) shifts of compounds **1**–**8**. Comparison between the hydrogens H-3, H-4, H-5 of the heterocycles, iminic, and amidic hydrogens in NAH-derivatives **1**–**8**, demonstrating the deshielding in selenophene isosters.

Compound	Ar	X	^1^H NMR Shift (ppm) 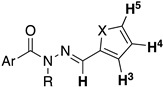				
			H-3	H-4	H-5	OCNH	N=CH
LASSBio-294 (**1**)	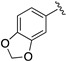	S	7.43–7.45	7.13	7.66	11.56	8.62
LASSBio-2062 (**3**)		Se	7.62	7.35	8.23	11.68	8.60
LASSBio-2092 (**4**)	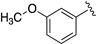	S	7.49–7.44	7.15	7.68	11.68	8.66
LASSBio-2093(**5**)		Se	7.62	7.36	8.24	11.85	8.68
LASSBio-785 (**2**)	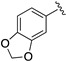	S	7.39	7.09	7.54	-	8.22
LASSBio-2063 (**6**)		Se	7.56	7.31	8.10	-	8.16
LASSBio-2198 (**7**)	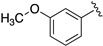	S	7.38	7.09	7.52	-	8.24
LASSBio-2199 (**8**)		Se	7.56	7.30	8.09	-	8.20

**Table 2:** Affinity and intrinsic efficacy of the *N*-acylhydrazone derivatives at the A_2A_ receptor (A_2A_R) present in membrane preparations from rat striatum. Average curves of three-four independent experiments (in triplicate) were analyzed by non-linear regression to estimate the IC_50_ values with their 95% confidence interval (95% CI). For each compound, the Na^+^-shifts were calculated by dividing the IC_50_ value obtained in the medium containing NaCl (II) by the IC_50_ value obtained in the medium containing MgCl_2_ (I) and statistically analyzed by paired t test on log IC_50_ values (P). Na^+^-shifts lower than one indicates that compounds **1** and **3**–**5** are weak inverse agonists, whereas Na^+^-shifts higher indicate that *N*-methylated compounds **2** and **6**–**8** are weak partial agonists.

Compound	IC_50_ (µM) MgCl_2_ (I)	IC_50_ (µM) NaCl (II)	Na^+^-Shift (II/I)	P
LASSBio-294 (**1**)	38.9 (33.1–45.4)	24.4 (22.0–27.1)	0.6	0.018
LASSBio-2062 (**3**)	20.9 (17.5–24.9)	11.3 (10.0–12.8)	0.5	0.010
LASSBio-2092 (**4**)	10.8 (9.6–12.2)	7.3 (6.4–8.4)	0.7	0.016
LASSBio-2093 (**5**)	10.4 (8.7–12.5)	6.7 (5.6–8.1)	0.6	0.011
LASSBio-785 (**2**)	47.9 (39.4–58.3)	107 (84–135)	2.3	0.043
LASSBio-2063 (**6**)	52.9 (43.4–64.4)	86.9 (70.6–107)	1.7	0.004
LASSBio-2198 (**7**)	22.3 (19.8–25.2)	54.6 (44.7–66.8)	2.5	0.010
LASSBio-2199 (**8**)	15.4 (13.4–17.6)	47.9 (37.0–61.9)	3.1	0.039

**Table 3:** Affinity and intrinsic efficacy of retroisosteric compounds **13** and **14** at the A_2A_R present in membrane preparations from rat striatum. Average curves of three independent experiments (in triplicate) were analyzed by non-linear regression to estimate the IC_50_ values with their 95% confidence interval (95% CI). For each compound, the Na^+^-shifts were calculated by dividing the IC_50_ value obtained in the medium containing Na^+^ by the IC_50_ value obtained in the medium containing Mg^2+^ and statistically analyzed by paired t test on log IC_50_ values (*P*).

Compound	IC_50_ (µM) MgCl_2_ (I)	IC_50_ (µM) NaCl (II)	Na^+^-Shift (II/I)	P
LASSBio-2278 (**13**)	27.1 (24.6–30.0)	55.6 (46.7–66.3)	2	0.010
LASSBio-2279 (**14**)	16.6 (15.3–18.1)	37.7 (34.5–41.2)	2.3	0.007

## Data Availability

Not applicable.

## Supplementary Materials

The following are available online, Synthetic description of intermediates **10a**–**b**, **11a**–**b**, **18**, and **19**; Figure S1: Na^+^-shift assay for the estimation of the intrinsic efficacy of the NAH derivatives **1**–**4**, **6**, and **7** at the A_2A_ receptor (A_2A_R) in rat striatum membrane preparation. Figure S2: Na^+^-shift assay for the estimation of the intrinsic efficacy of the retroisosteric analogues at the A_2A_ receptor (A_2A_R) in rat striatum membrane preparation. Figures S1–S18: NMR and HRMS spectra of compounds **3**–**8**, **11**, and **12**; Table S1: ^13^C NMR shifts of the iminic hydrogen of the NAH in isosteric compounds **1**–**8**.
