# Supplementary material for: Effect of S–Se Bioisosteric Exchange on Affinity and Intrinsic Efficacy of Novel N-acylhydrazone Derivatives at the Adenosine A2A Receptor
Source: Molecules. 2021 Dec 4;26(23):7364. doi: 10.3390/molecules26237364 (PMC8659164; doi:10.3390/molecules26237364)
Supplement: Supplementary file 1 [file molecules-26-07364-s001.zip › molecules-1454274-supplementary/molecules-1454274-supplementary.pdf]

Conformational effect on affinity and intrinsic efficacy of novel *N*-acylhydrazone derivatives in A_{2A} adenosinergic receptor.

Júlia G. B. Pedreira[a,b†], Rafaela R. Silva[c†], François Noël[c,d], Eliezer J. Barreiro[a,b,d]

[a]Laboratory of Evaluation and Synthesis of Bioactive Substances (LASSBio), Institute of Biomedical Sciences, Federal University of Rio de Janeiro (UFRJ), 21944-971, Rio deJaneiro, RJ, Brazil.

[b]Graduate Program of Chemistry (PGQu), Chemistry Institute, UFRJ, 21941-909, Rio de Janeiro, RJ, Brazil

[c]Laboratory of Biochemical and Molecular Pharmacology, Federal University of Rio de Janeiro (UFRJ), 21944-971, Rio de Janeiro, RJ, Brazil.

[d] Research Program in Drug Development (PPDF), Institute of Biomedical Sciences, FederalUniversity of Rio de Janeiro (UFRJ), 21944-971, Rio de Janeiro, RJ, Brazil.

[†] These two authors contributed equally to this work

Table of contents

1. General information… 2

2. Synthesis and characterization of intermediates (**16a-b**, **9a-b**, **13** and **14**).......................... 2

3. Na^+-shift curves for *N*-acylhydrazone compounds **1-4**, **6**, **7**, **11** and **12** 6

4. NMR spectra of compounds **3-8, 11** and **12**.. 7

5. References 23

1. General information

All reagents and (anhydrous) solvents are commercially available and were used without further purification. NMR spectra were obtained at UFRJ with a VARIAN 400-MR and 500-MR (IPPN-UFRJ). The spectra were obtained in the indicated solvent and calibrated against the residual proton peak of the deuterated solvent. Chemical shifts (δ) are reported in parts per million. Mass spectra were obtained on an Esquire 6000-ESI Ion Trap MSn System Bruker Daltonics (LASSBio-UFRJ).

2. Synthesis and characterization of intermediates (16a-b, 9a-b, 13 and 14)

*2.1. Synthesis of ester intermediates (**16a** and **16b**)*[1]

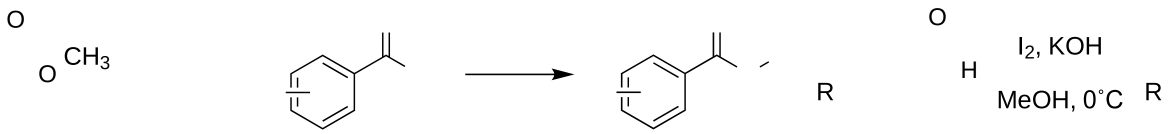

16a (R = 3,4-(OCH_2O))
16b (R = 3-OCH_3)

15a (R = 3,4-(OCH_2O))
15b (R = 3-OCH_3)

The corresponding aldehyde (**15a** and **15b**) was solubilised in MeOH (0.2 M) in a round-bottom flask. A solution of KOH in MeOH (3 eq., 0.8 - 1.3 M) was added and the flask was cooled on an ice bath at 0˚C. Then, a solution of I_2 in MeOH (1.5 eq., 0.3 - 0.5 M) was slowly added dropwise, and the reaction stirred at room temperature. TLC control was made (20% EtOAc/*n*-hex) until the full conversion of the aldehyde (max. 4 h). The quenching was made by reduction of the volume of MeOH on a rotary evaporator followed by dropwise addition of a saturated solution of $NaHSO_3$ (*ca* 20 mL) until the reaction mixture turned to white. The isolation of the product was made by either filtration under vacuum on Büchner funnel or extraction of the aqueous phase with EtOAc (3 x 15 mL) followed by drying over Na_2SO_4 and removal of the solvent with a rotary evaporator.

2.1.1. *Methyl benzo[d][1,3]dioxole-5-carboxylate (**16a**)*

The intermediate **16a** was prepared according to procedure described on item 2.1, to afford a white solid with 77% yield (3.6 g, 20 mmol) with m.p. 50˚C (Lit. 53˚C).[2]

^{1}H NMR (400 MHz, DMSO) δ: 7,57 (d, *J* = 6,8 Hz, 1H, H6), 7,38 (s, 1H, H2), 7,04 (d, *J* = 8,2 Hz, 1H, H5), 6,14 (s, 2H, O-C<u>H</u>2-O), 3,81 (s, 3H, OC<u>H</u>3).

*2.1.2. Methyl 3-methoxybenzoate (**16b**)*

The intermediate **16b** was prepared according to the procedure described on item 2.1, to afford a brown oil with 79% yield (0.34 g, 2.0 mmol).[3]

^{1}H NMR (400 MHz, DMSO) δ: 7.41 – 7.33 (m, 3H, H2, H4 and H6), 7.07 – 7.05 (m, 1H, H2), 3,78 (s, 3H).

2.2. Synthesis of hydrazide intermediates (9a, 9b)[4]

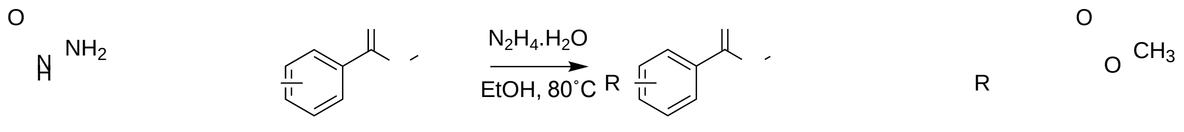

9a (R = 3,4-(OCH2O))
9b (R = 3-OCH3)

16a (R = 3,4-(OCH2O))
16b (R = 3-OCH3)

The corresponding ester (**16a, 16b**) was solubilised in EtOH (0.2 or 0.3 M), and hydrazide hydrate (80% in H_2O) was added (10 eq.). The flask was attached to a reflux condenser and the reaction was heated to reflux temperature and stirred for *ca.* 24h, untilTLC control (50 or 70% EtOAc/*n*-hexane) showed full conversion of the starting material. The volume of EtOH was reduced in a rotary evaporator and then water was added. The product was either filtrated under vacuum on a Büchner funnel or extracted with EtOAc,dried over Na_2SO_4 and the solvent evaporated on a rotary evaporator.

*2.2.1. Benzo[d][1,3]dioxole-5-carbohydrazide (**9a**)*

The intermediate **9a** was prepared according to procedure described on item 2.2 to afford a white solid with 80% yield (0.79 g, 4.4 mmol) with m.p. of 172˚C (Lit. 176˚C).[5]

^{1}H NMR (500 MHz, DMSO) δ: 9,60 (s, 1H, N<u>H</u>), 7,41 (dd, *J* = 8,1, 1,7 Hz, 1H, H6), 7,35 (s, 1H, H2), 6,97 (d, *J* = 8,1 Hz, 1H, H5), 6,08 (s, 2H, O-C<u>H</u>$_2$-O), 4,42 (s, 2H, N<u>H</u>$_2$). MS: m/z 181 $[M+1]^+$

*2.2.2. 3-Methoxybenzohydrazide (**9b**)*

The intermediate **9b** was prepared according to the procedure described on item 2.2, to afford a brown oil with 93% yield (0.32 g, 1.9 mmol).[6]

^{1}H NMR (400 MHz, DMSO) δ: 9.74 (s, 1H, OCN<u>H</u>NH$_2$), 7.41 - 7.33 (m, 3H, H2, H4 and H6), 7.07 - 7.05 (m, 1H, H5), 4.45 (bs, 2H, OCNHN<u>H</u>$_2$), 3.78 (s, 3H, OCH_3).

MS: m/z 167 $[M+1]^+$

*2.2.3. Thiophene-2-carbohydrazide (**13**)*

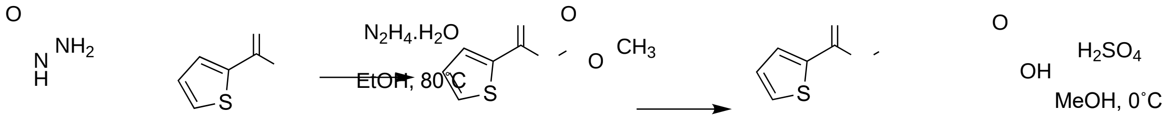

17 **18** **13**

Intermediate **13** was obtained in two steps. The carboxylic acid **17** (1 g, 7.8 mmol) was solubilised in MeOH (0.2 M), and a 2% v/v H_2SO_4 in MeOH solution was added.[7] The reaction was stirred at reflux temperature overnight (18h) until full conversion was observed in TLC control (20% EtOAc/*n*-hex). The reaction was allowed to cool down to room temperature and quenched to pH ~7 with solid Na_2CO_3. The organic solvent was evaporated on a rotary evaporator and water was added. The product was extracted with EtOAc (3 x 20 mL), dried over $NaHSO_4$ and the organic phase evaporated on a rotary evaporator to afford ester intermediate **18**[8] as a colourless oil. The following hydrazinolysis reaction was performed by the procedure described on item 2.2, to afford **13** as a white solid with 70% yield (0.77 g, 5.4 mmol).[9]

^{1}H NMR (400 MHz, DMSO-d_6) δ 9.7 (s, 1H), 7.73 – 7.70 (m, 2H), 7.12 (t, 1H, *J* = 7.2 Hz), 4.46 (s, 2H). ^{13}C NMR (100 MHz, DMSO-d_6) δ 161.3, 138.4, 130.3, 127.9, 127.5. MS: m/z 143 $[M+1]^+$

2.2.4. Selenophene-2-carbohydrazide (14)

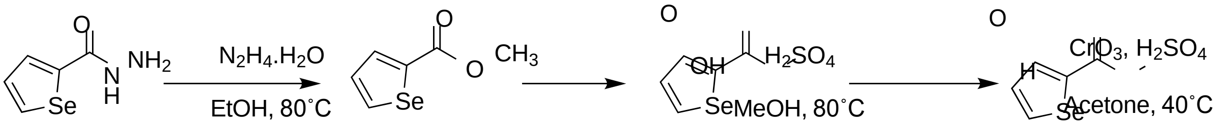

19 **20** **21** **14**

Intermediate **14** was obtained in a three-step synthesis. Preparation began with the Jones oxidation.[10] The aldehyde **18** (250 mg, 1.57 mmol) was solubilised in acetone (0.2 M) in a round-bottom flask. The reaction flask was put on an ice bath, and a solution of Jones reagent(1 eq., 1.94 g/mL) was added dropwise, which turned the reaction to orange. The reaction mixture was stirred at *ca.* 30˚C for 24 and monitored via TLC (20% EtOAc/*n*-hex). The completion of the reaction can be observed by a change of colour from orange to green. Afterthe reaction was done, the green precipitate was filtrated and washed with acetone. The organic phase was evaporated, and the product adsorbed on Celite®. Purification was doneby flash chromatography (0 - 30% EtOAc/n-hex) to afford **20** a white solid (240 mg, 1.36 mmol) with 86% yield.[11] The carboxylic acid **20** was then solubilised in MeOH (0.5 M) and a2% v/v solution of H_2SO_4 in MeOH was added.[7] The reaction was stirred at 60˚C for 24huntil full conversion was observed in TLC control 20% (EtOAc/*n*-hex). The reaction was quenched to pH ~7 with solid $NaHCO_3$ and the organic solvent evaporated on a rotary

evaporator. Water was added and the product was extracted with EtOAc (3 x 10 mL), dried over Na_2SO_4 and the organic phase evaporated on a rotary evaporator to afford ester intermediate **21** as a light yellow oil.[12] The hydrazide intermediate **22** was subsequently obtained by the general procedure described on item 2.2 to afford a white solid (0.14 g, 0.74 mmol) with 75% yield.[13]

^{1}H NMR (400 MHz, DMSO-d_6) δ 9.75 (s, 1H), 8.35 (dd, 1H, J_1 = 5.5 Hz, J_2 = 1 Hz), 7.89 (dd, 1H, J_1 = 3.9 Hz, J_2 = 1 Hz), 7.36 (dd, 1H, J_1 = 5.5 Hz, J_2 = 3.9 Hz), 4.53 (s, 2H). ^{13}C NMR (150 MHz, DMSO-d_6) δ 162.9, 148.4, 137.0, 130.9, 130.0. MS: m/z 190 $[M+1]^+$

*2.3. ^{13}C NMR shifts of iminic carbons of the NAH moiety of compounds **1-8**.*

Table S1: ^{1}H NMR (400 MHz, DMSO-d^6, 25˚C) shifts of the iminic hydrogen in the NAH of compounds **1-8**.

**Compound**	Ar	**X**	**^{1}H NMR shift (ppm)** N=C̲H
LASSBio-294 (**1**)	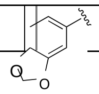	S	139.2
LASSBio-2062 (**3**)		Se	143.9
LASSBio-2092 (**4**)	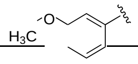	S	143.1
LASSBio-2093(**5**)		Se	145.0
LASSBio-785 (**2**)	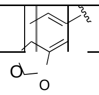	S	136.0
LASSBio-2063 (**6**)		Se	137.1
LASSBio-2198 (**7**)	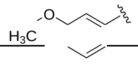	S	N.D.
LASSBio-2199 (**8**)		Se	146.0

3. Na+-shift curve for *N*-acylhydrazone compounds 1-4, 6, 7, 11 and 12.

A

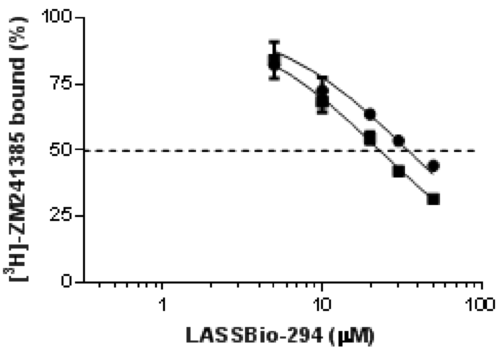

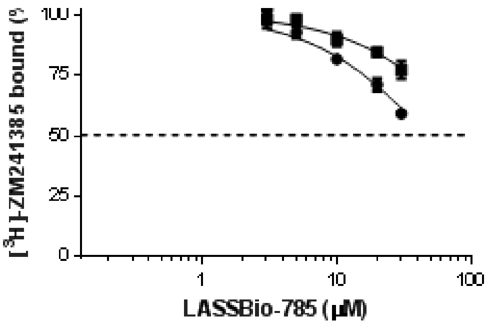

B

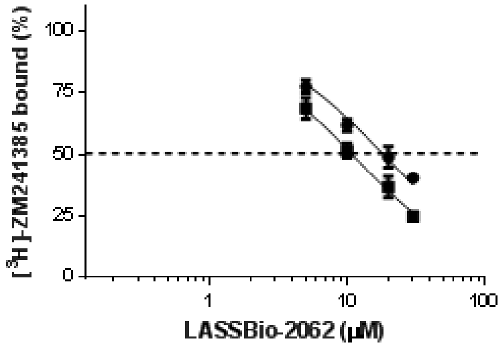

C

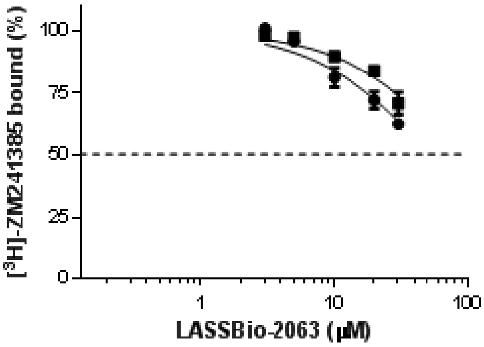

D

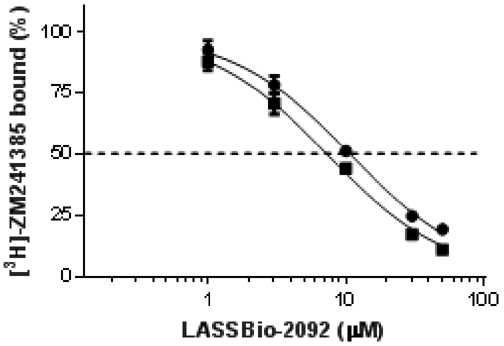

E

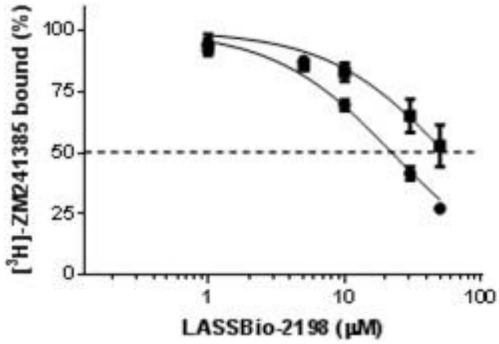

F

Figure S1. Na^+-shift assay for estimation of the intrinsic efficacy of the NAH derivatives **1-4**, **6**, and **7** at the A_{2A} receptor ($A_{2A}R$) in rat striatum membrane preparation. Competition curves were performed using the antagonist radioligand (0.5 nM [^{3}H]-ZM241385) in the presence of 50 mM $MgCl_2$ (circle) or 100 mM NaCl (square). Each curve represents the averaged curve (±S.E.) from three independent paired experiments (in triplicate). Curves were drawn using the parameters fitted by nonlinear regression (“One site competition”, GraphPad Prism version 6.0®). (**A**) LASSBio-294 (**1**); (**B**) *N*- methylated LASSBio-785 (**2**); (**C**) LASSBio-2062 (**3**); (**D**) *N*-methylated LASSBio-2063 (**6**);(**E**) LASSBio-2092 (**4**); (**F**) *N*-methylated LASSBio-2198 (**7**).

A **B**

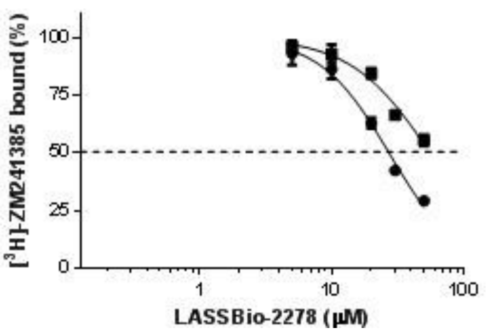

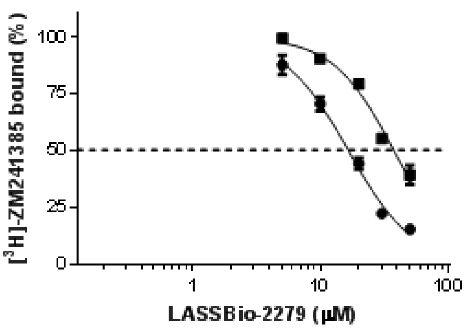

Figure S2. Na+-shift assay for estimation of the intrinsic efficacy of the retroisosteric analogues at the A_{2A} receptor (A_{2A}R) in rat striatum membrane preparation. Competition curves were performed using the antagonist radioligand (0.5 nM [^{3}H]-ZM241385) in the presence of 50 mM $MgCl_2$ (circle) or 100 mM NaCl (square). Each curve represents the averaged curve (±S.E.) from three independent paired experiments (in triplicate). Curves were drawn using the parameters fitted by nonlinear regression ("log(inhibitor) vs. response - Variable slope", GraphPad Prism version 6.0®). (**A**) LASSBio-2278 (**11**); (**B**) *N*-methylated LASSBio-2279 (**12**).

4. NMR spectra of compounds 3-8, 11 and 12

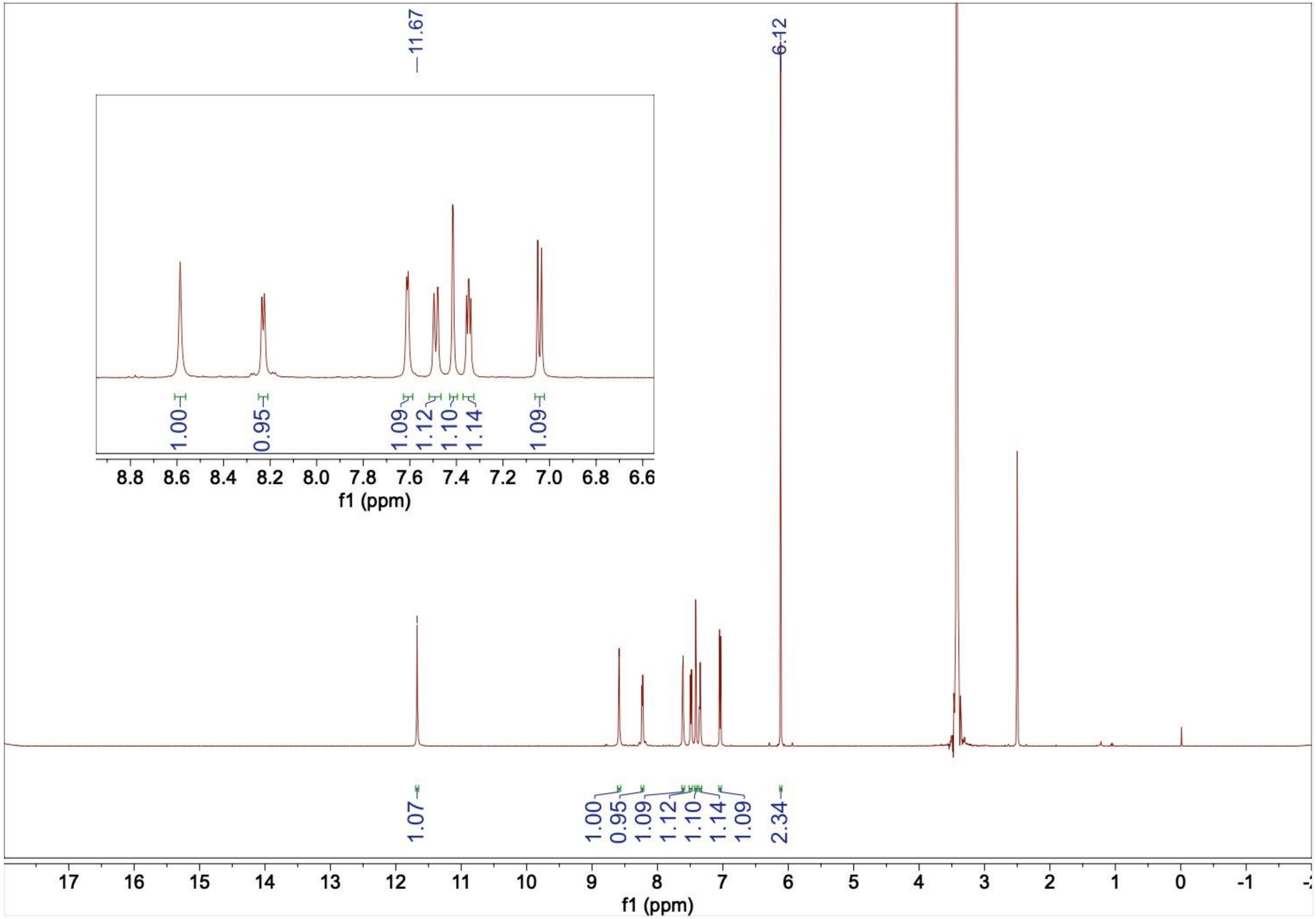

Figure S**3.** ^{1}H NMR spectrum (400 MHz, DMSO-d^{6}, 25˚C): *N'*-(selenophen-2-ylmethylene)benzo[d][1,3]dioxole-5-carbohydrazide (**3**, LASSBio-2062).

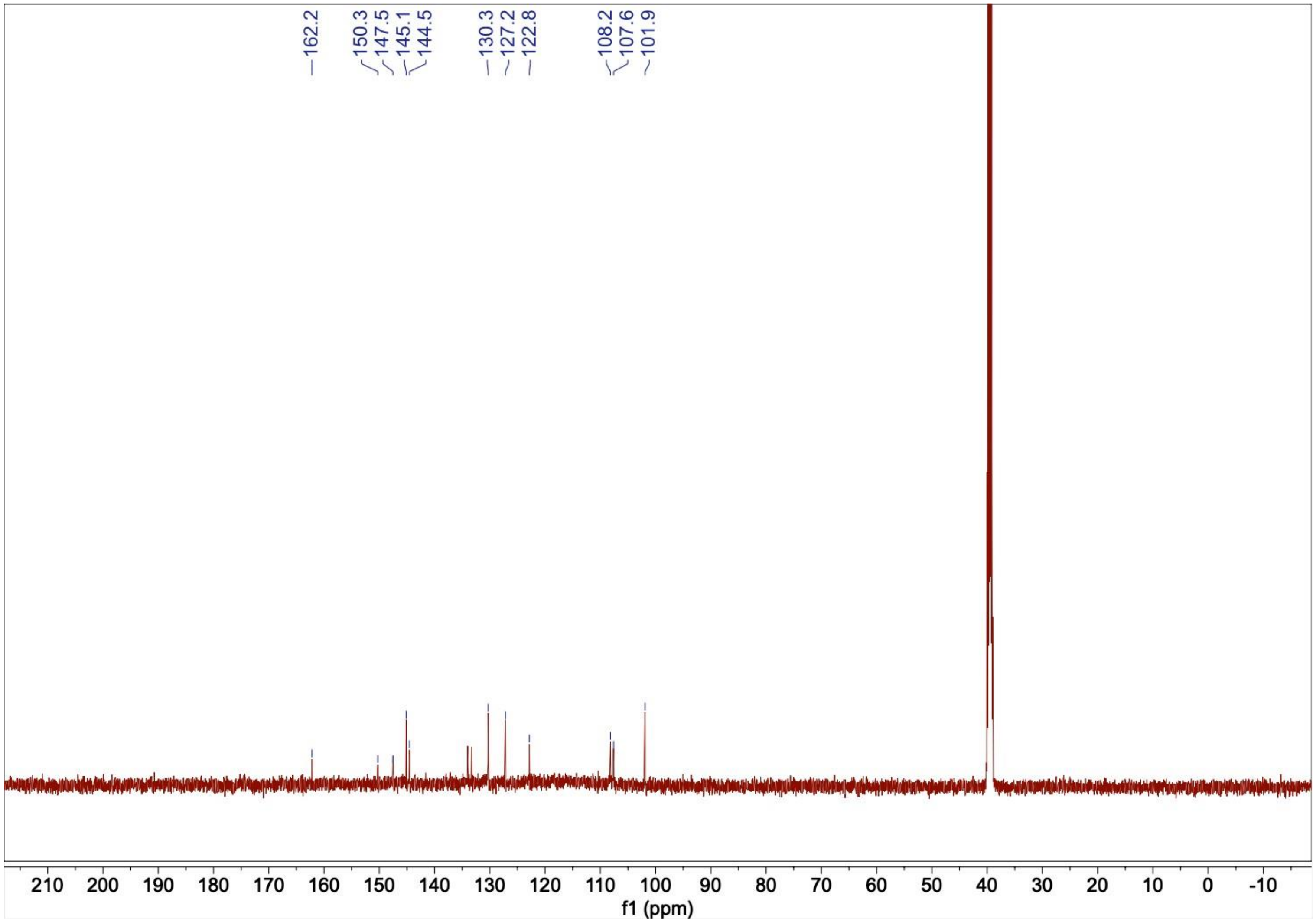

Figure S4. ^{13}C NMR spectrum (100 MHz, DMSO-d^6, 25˚C): *N'*-(selenophen-2-ylmethylene)benzo[d][1,3]dioxole-5-carbohydrazide (**3**, LASSBio-2062).

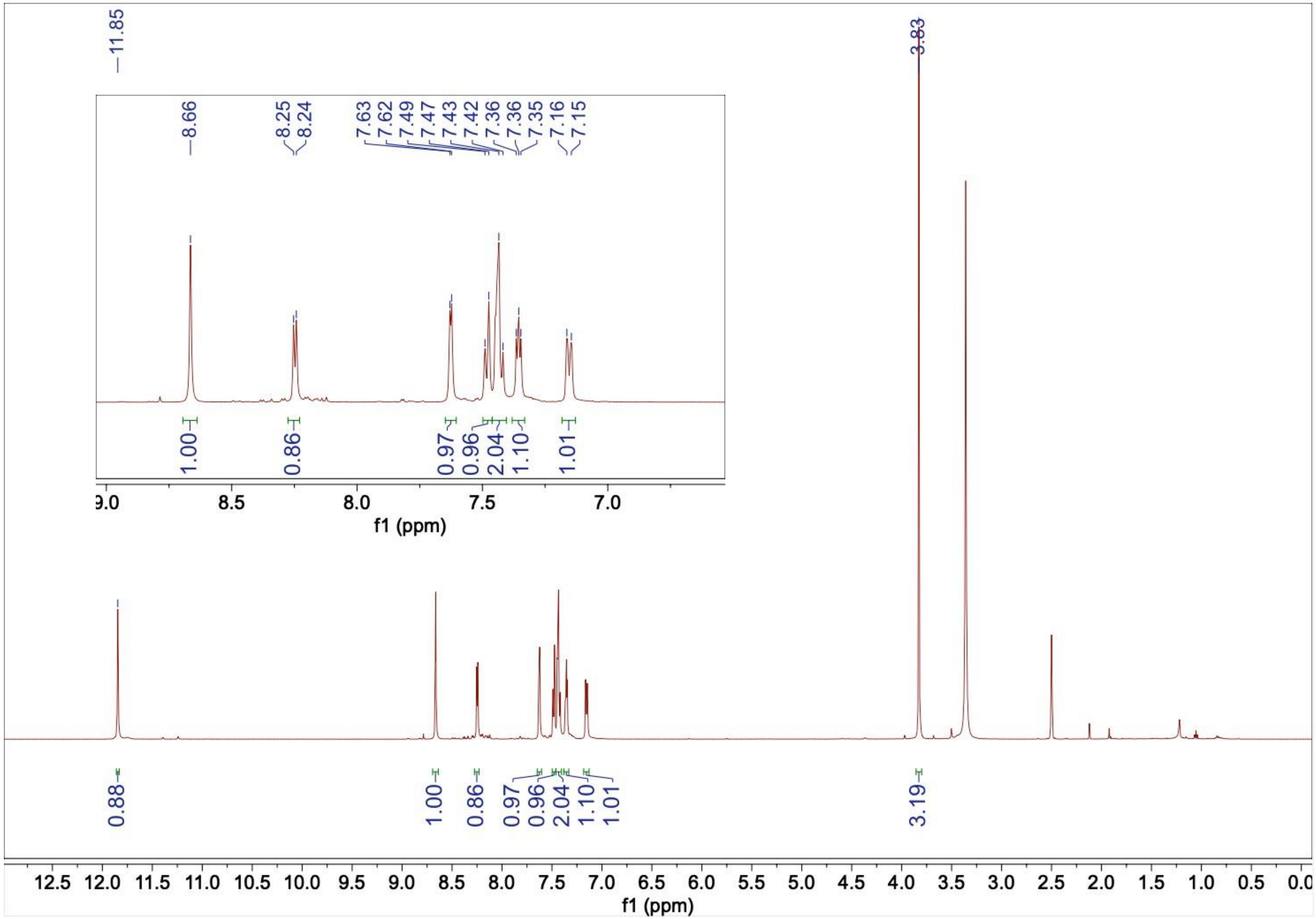

Figure S**5.** ^{1}H NMR spectrum (400 MHz, DMSO-d^{6}, 25˚C): 3-methoxy-*N*'-(thiophen-2-ylmethylene)benzohydrazide (**4**, LASSBio-2092).

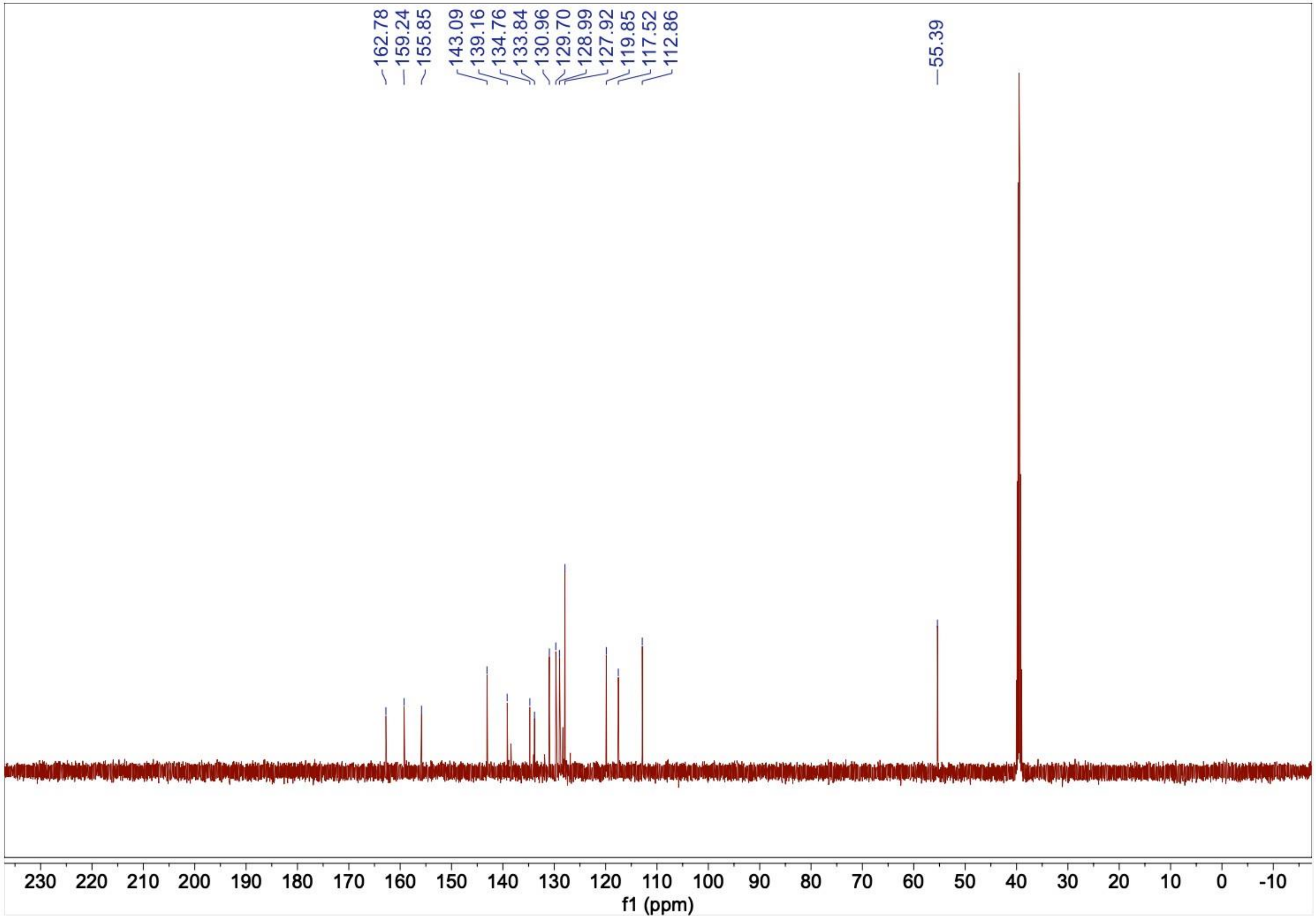

Figure S**6.** ^{13}C NMR spectrum(400 MHz, DMSO-d^6, 25˚C) : 3-methoxy-*N*'-(thiophen-2-ylmethylene)benzohydrazide (**4**, LASSBio-2092).

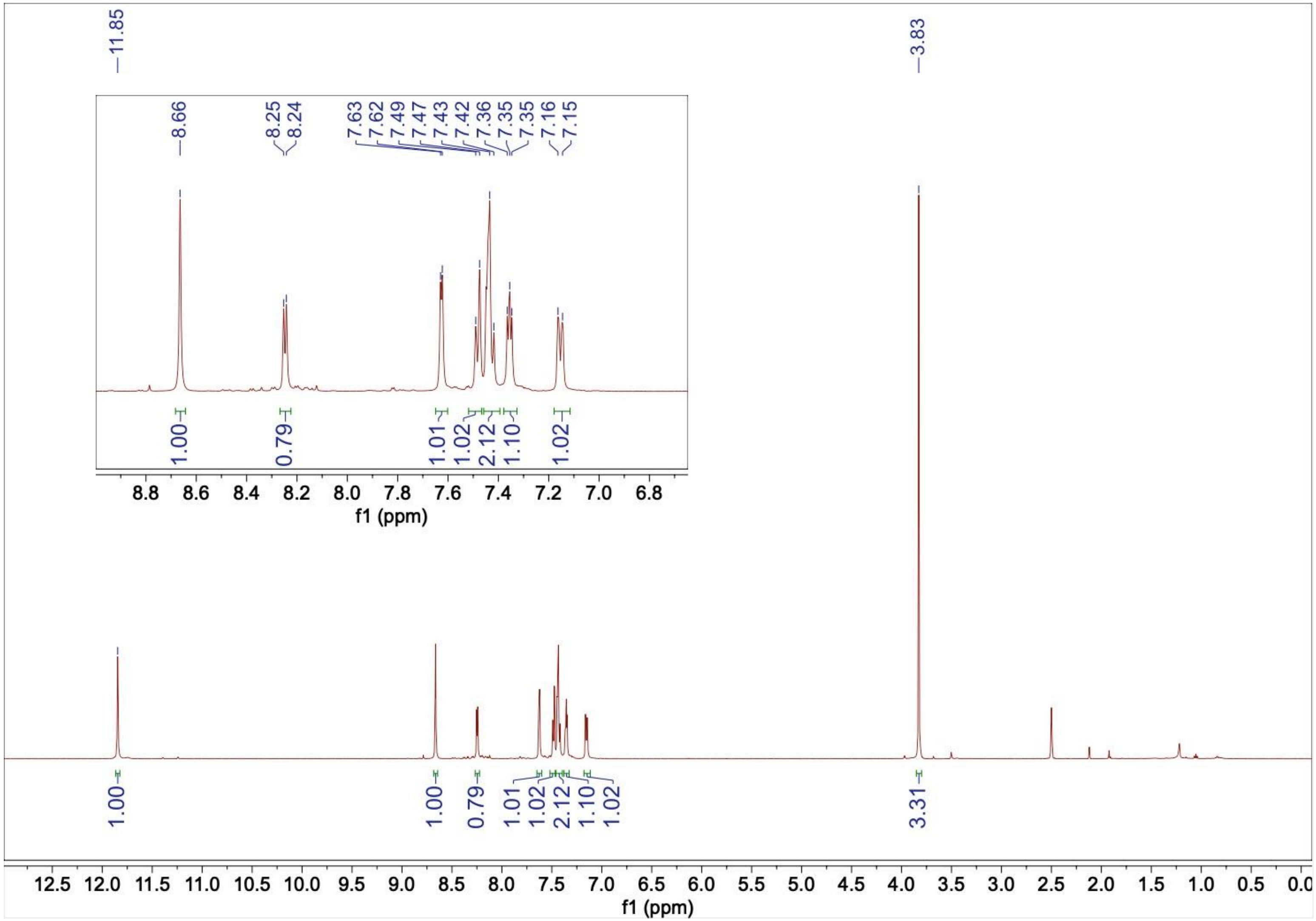

Figure S7. ^{1}H NMR spectrum (400 MHz, DMSO-d^6, 25˚C): 3-methoxy-*N'*-(thiophen-2-ylmethylene)benzohydrazide (**5**, LASSBio-2093).

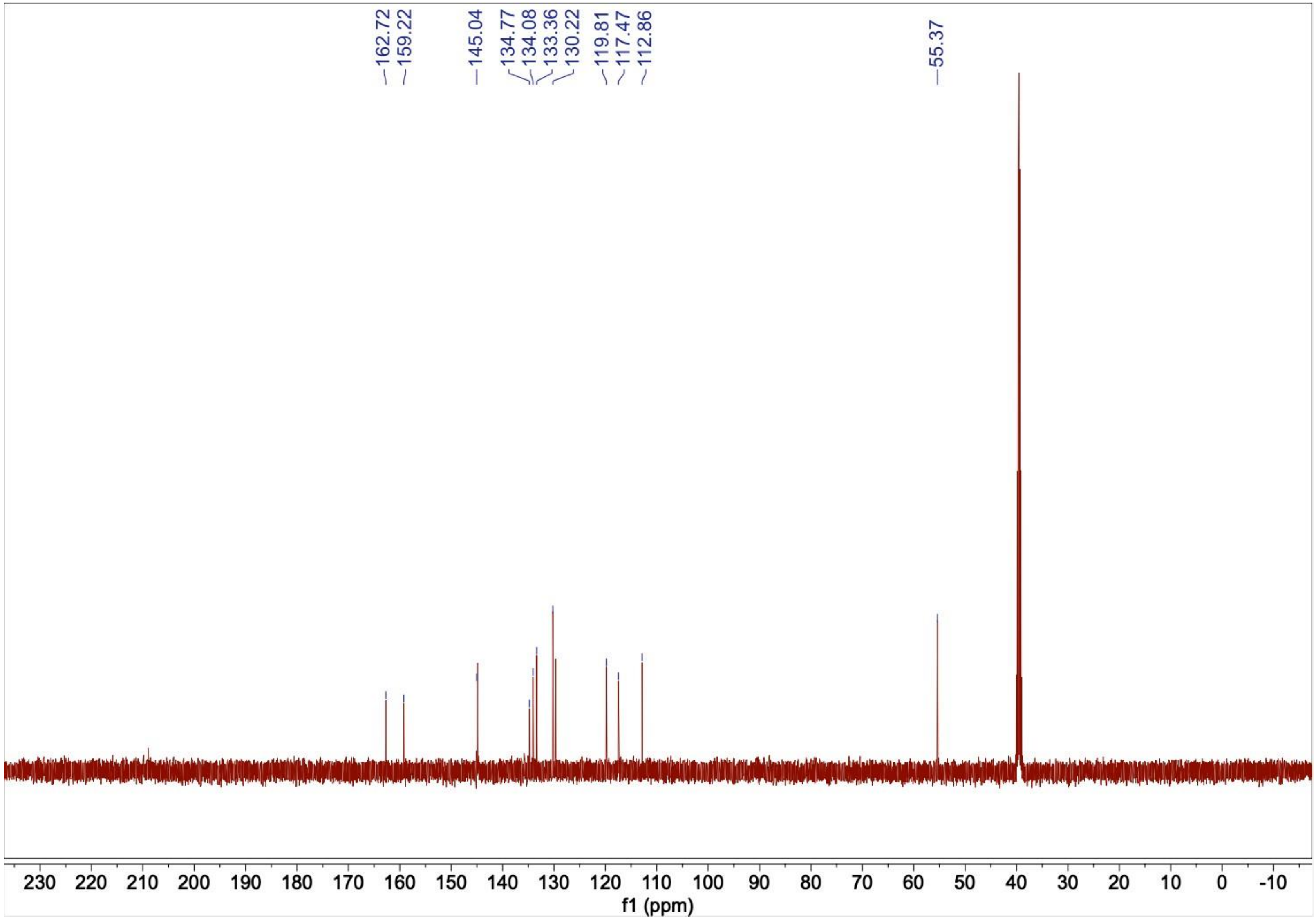

Figure S8. ^{13}C NMR spectrum (100 MHz, DMSO-d^{6}, 25˚C): 3-methoxy-*N*'-(thiophen-2-ylmethylene)benzohydrazide (**5**, LASSBio-2093).

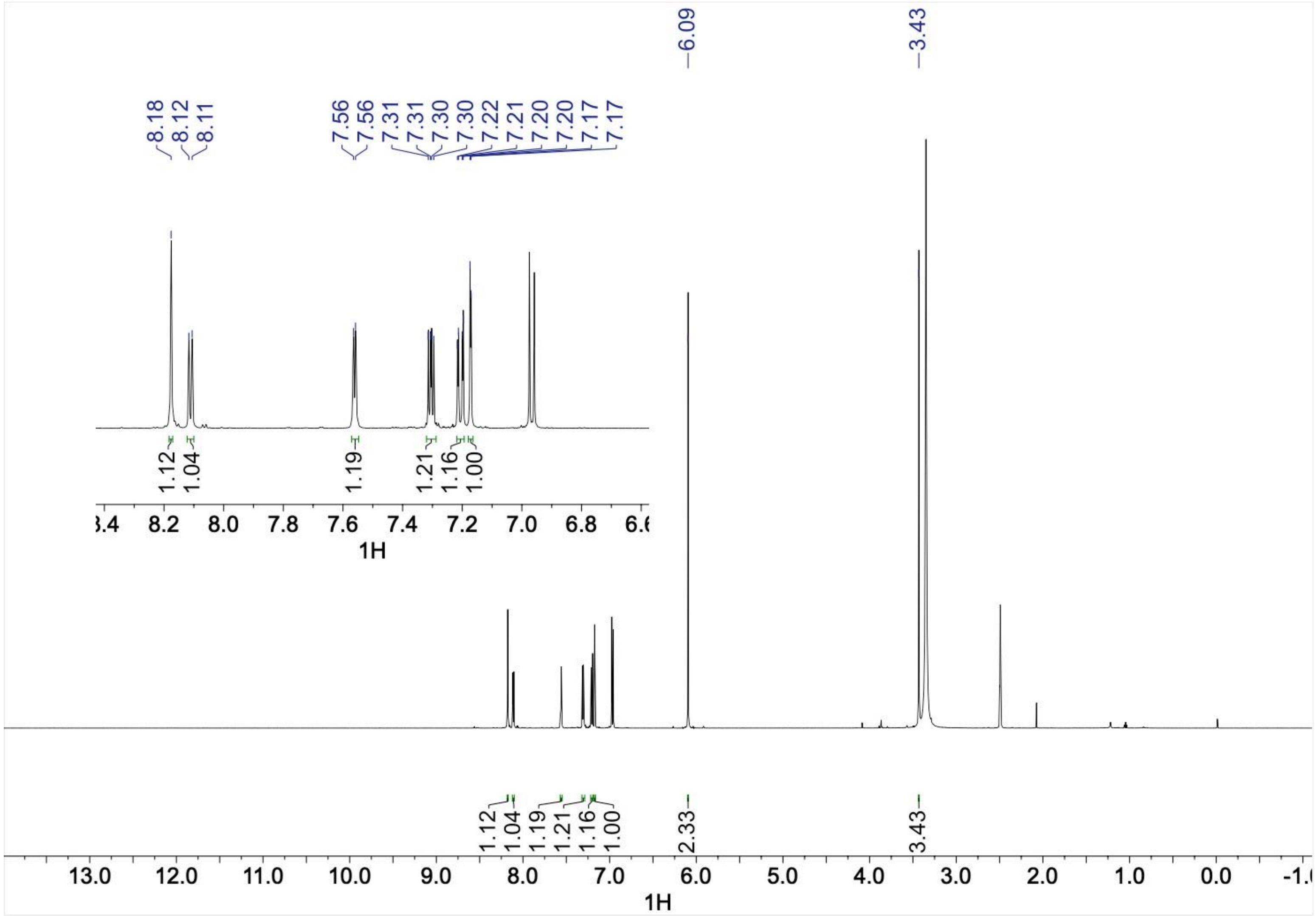

Figure S9. ^{1}H NMR spectrum (400 MHz, DMSO-d^6, 25˚C): *N*-methyl-*N*'-(selenophen-2-ylmethylene)benzo[d][1,3]dioxole-5-carbohydrazide (**6**, LASSBio-2063).

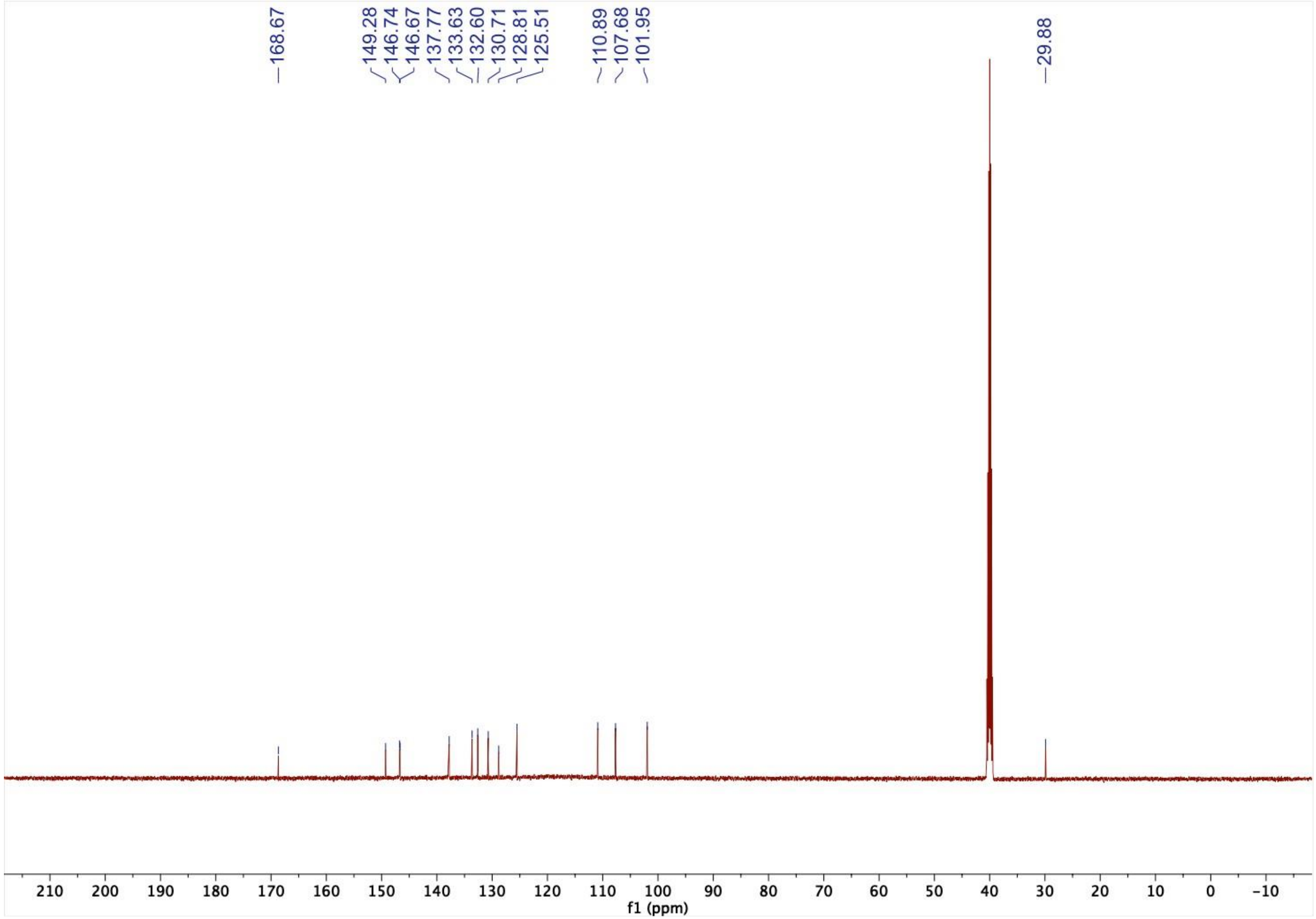

Figure S**10.** ^{13}C NMR spectrum: *N*-methyl-*N*'-(selenophen-2-ylmethylene)benzo[d][1,3]dioxole-5-carbohydrazide (**6**, LASSBio-2063).

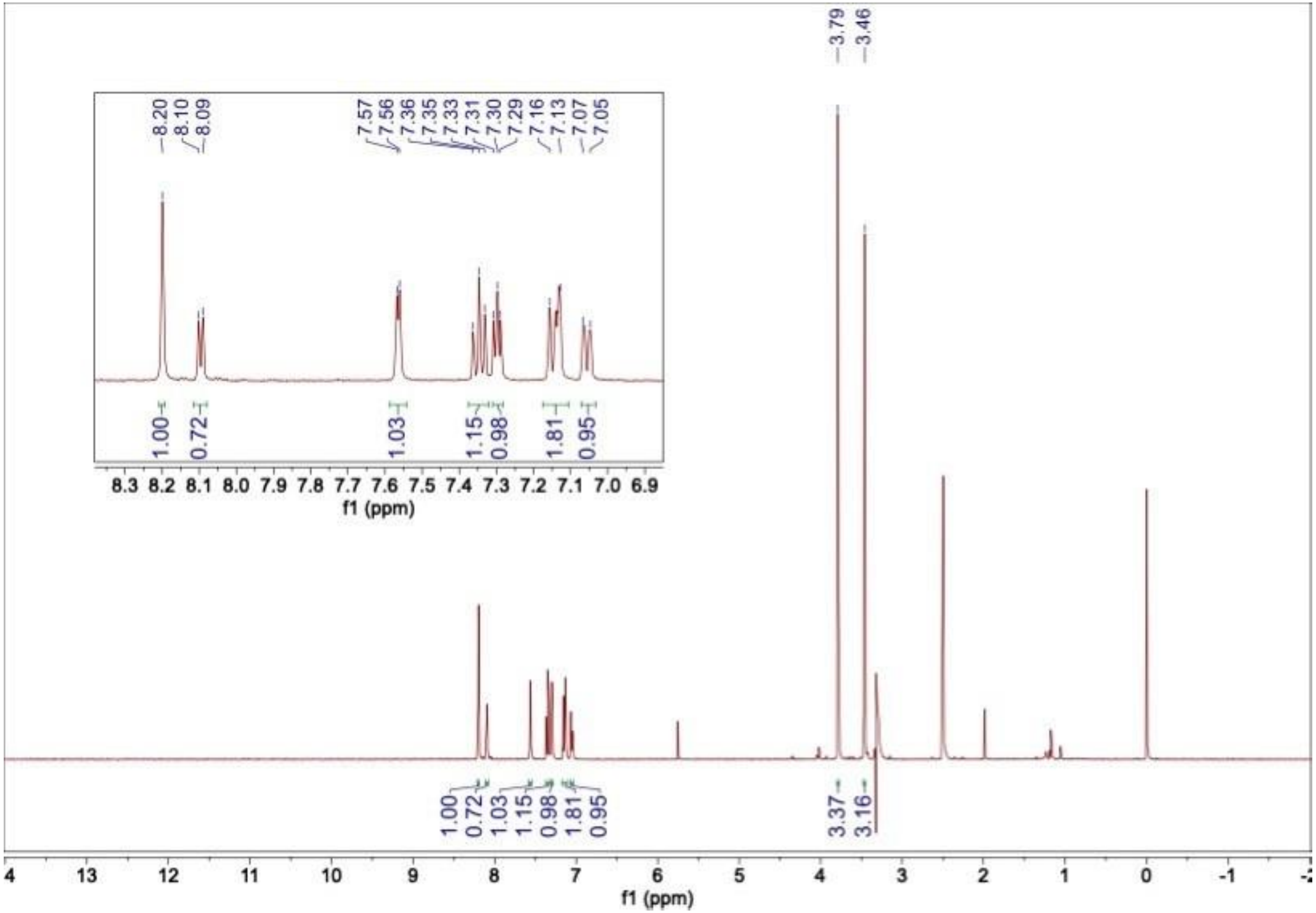

Figure S**11.** ^{1}H NMR spectrum (400 MHz, DMSO-d^{6}, 25˚C): 3-methoxy-*N*-methyl-*N*'-(thiophen-2-ylmethylene)benzohydrazide (**7**, LASSBio-2198).

==== Shimadzu LCsolution Analysis Report ====

C:\LabSolutions\Data\Dados HPLC\Julia\21092018\3OMe_NCH3_S.lcd

Acquired by	: Admin
Sample Name	: 3OMe_NCH3_S
Sample ID	: 3OMe_NCH3_S
Tray#	: 1
Vail #	: 9
Injection Volume	: 10 uL
Data File Name	: 3OMe_NCH3_S.lcd
Method File Name	: MET_60ACN_40AGUA_T20.lcm
Batch File Name	: tabela_21092018.lcb
Report File Name	: Default.lcr
Data Acquired	: 9/21/2018 10:54:37 AM
Data Processed	: 9/21/2018 11:14:38 AM

<Chromatogram>

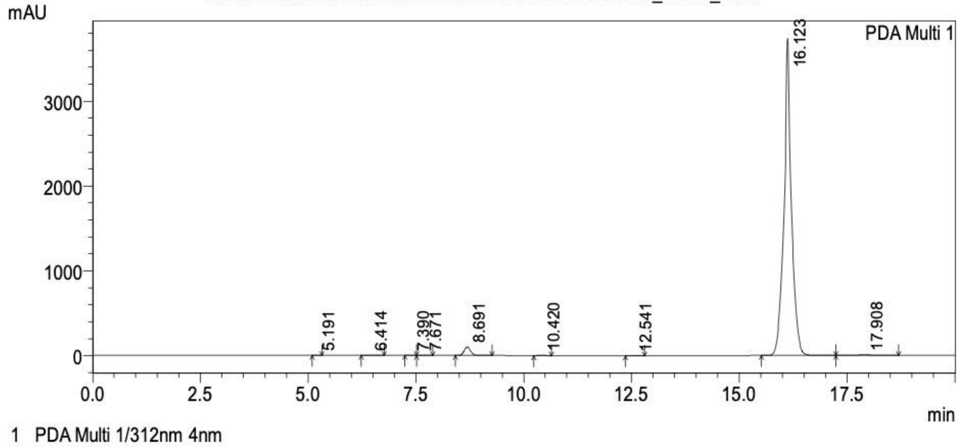

1 PDA Multi 1/312nm 4nm

PeakTable

PDA Ch1 312nm 4nm

Peak#	Ret. Time	Area	Height	Area %	Height %
1	5.191	1291	176	0.003	0.005
2	6.414	10596	1150	0.023	0.030
3	7.390	4226	364	0.009	0.009
4	7.671	16325	1562	0.035	0.041
5	8.691	1064736	101931	2.301	2.648
6	10.420	2907	266	0.006	0.007
7	12.541	2029	166	0.004	0.004
8	16.123	44951807	3735746	97.146	97.037
9	17.908	218613	8448	0.472	0.219
Total		46272529	3849809	100.000	100.000

Figure S**12.** HPLC Chromatogram of 3-methoxy-*N*-methyl-*N'*-(thiophen-2-ylmethylene)benzohydrazide(**7**, LASSBio-2198).

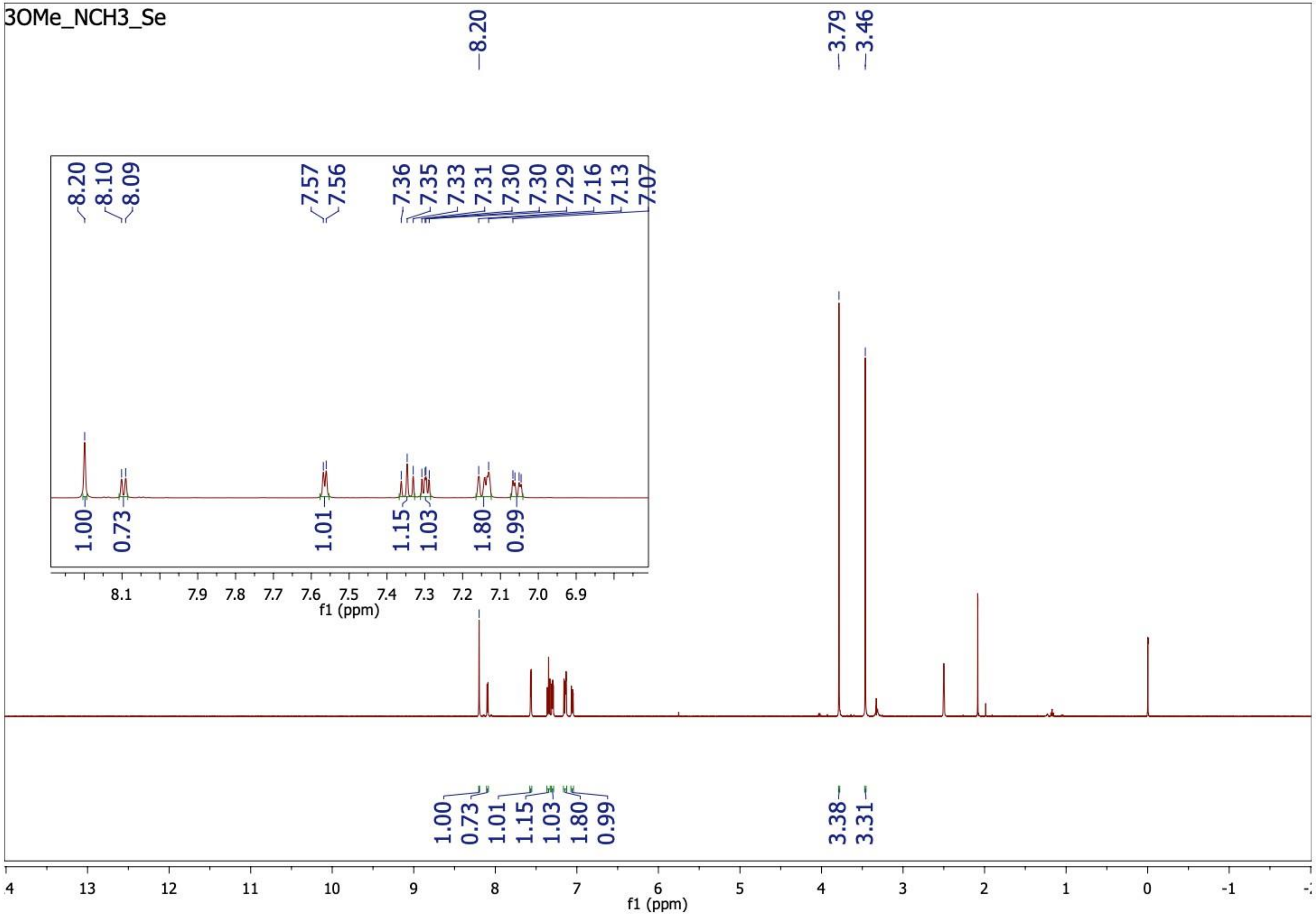

Figure S**13.** ^{1}H NMR spectrum (400 MHz, DMSO-d^{6}, 25˚C): 3-methoxy-*N*-methyl-*N*'-(selenophen-2-ylmethylene)benzohydrazide (**8**, LASSBio-2199,).

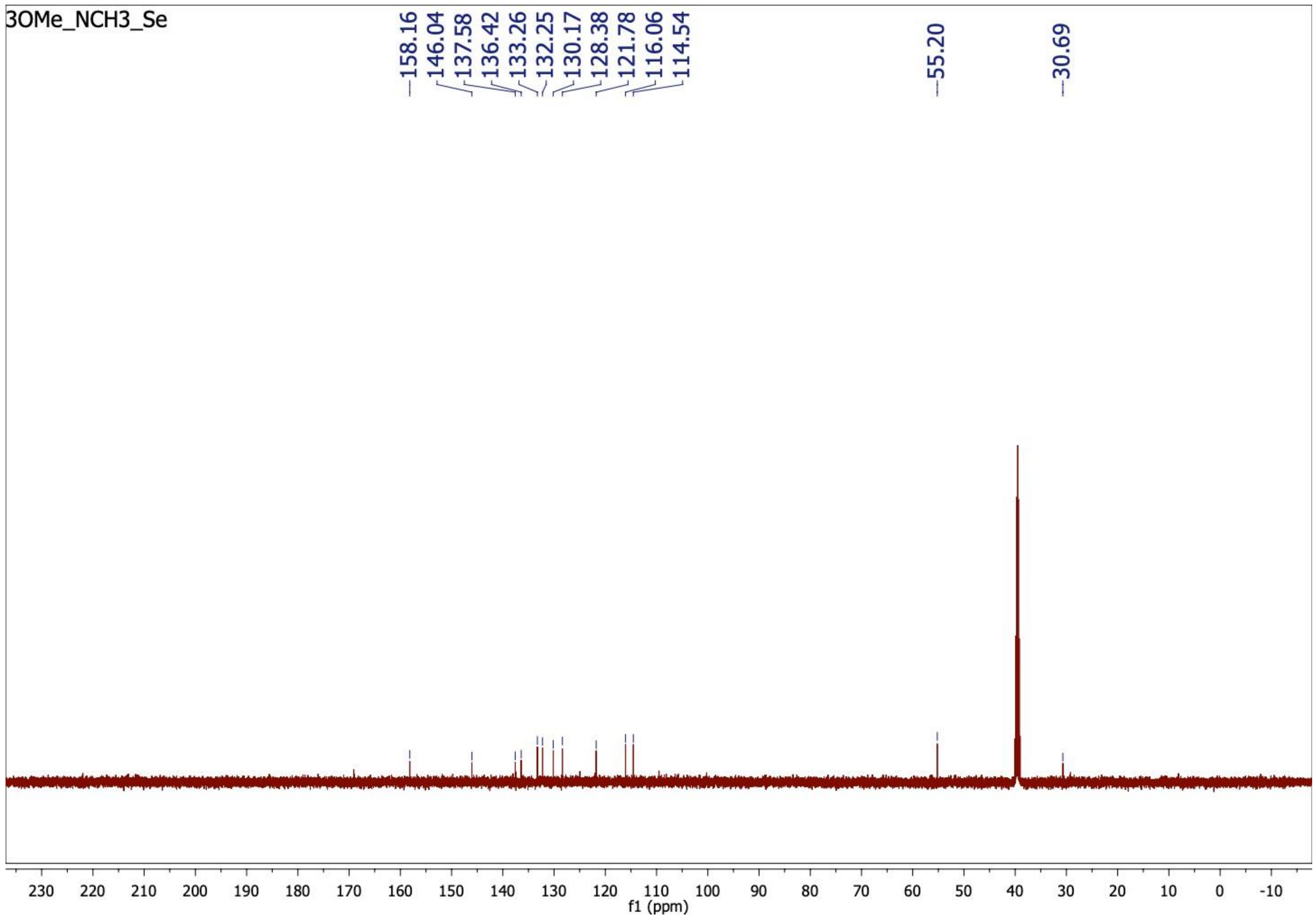

Figure S**14.** ^{13}C NMR spectrum (100 MHz, DMSO-d^6, 25˚C): 3-methoxy-*N*-methyl-*N*'-(selenophen-2-ylmethylene)benzohydrazide (**8**, LASSBio-2199).

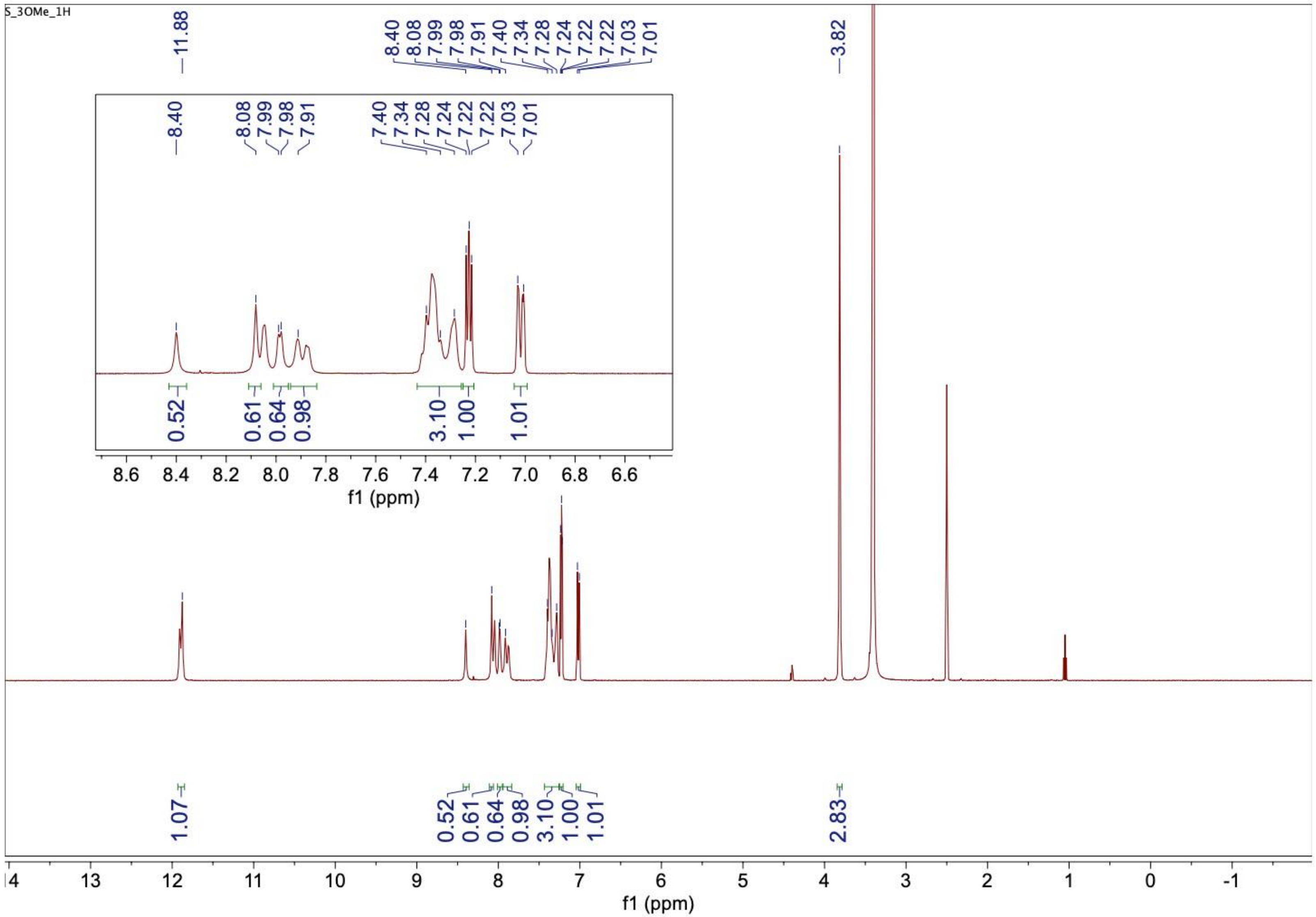

Figure S**15.** ^{1}H NMR spectrum (400 MHz, DMSO-d^{6}, 25˚C): *N'*-(3-methoxybenzylidene)thiophene-2-carbohydrazide (**11**, LASSBio-2278).

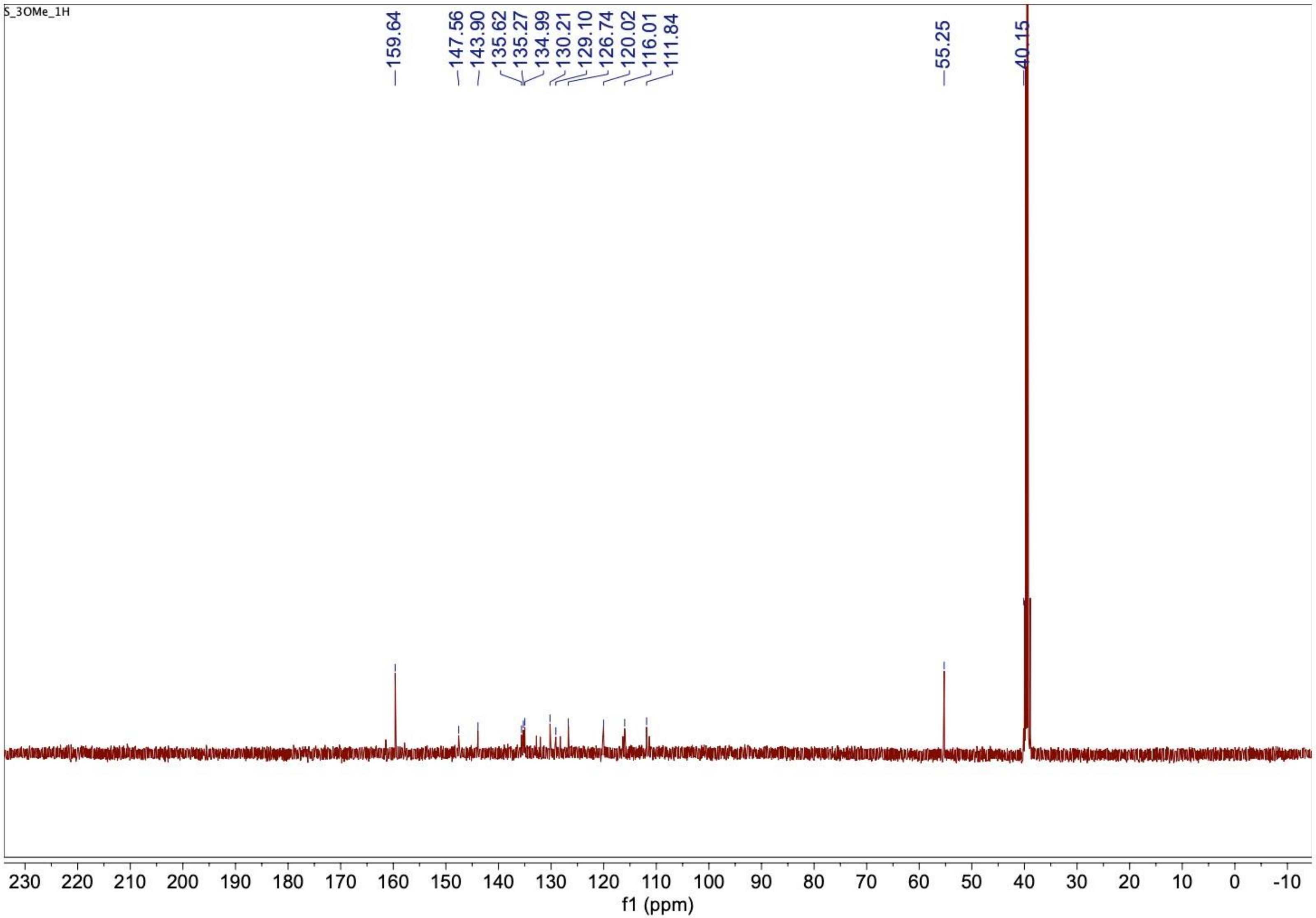

Figure S16. ^{13}C NMR spectrum (100 MHz, DMSO-d^6, 25˚C): *N'*-(3-methoxybenzylidene)thiophene-2-carbohydrazide (**11**, LASSBio-2278).

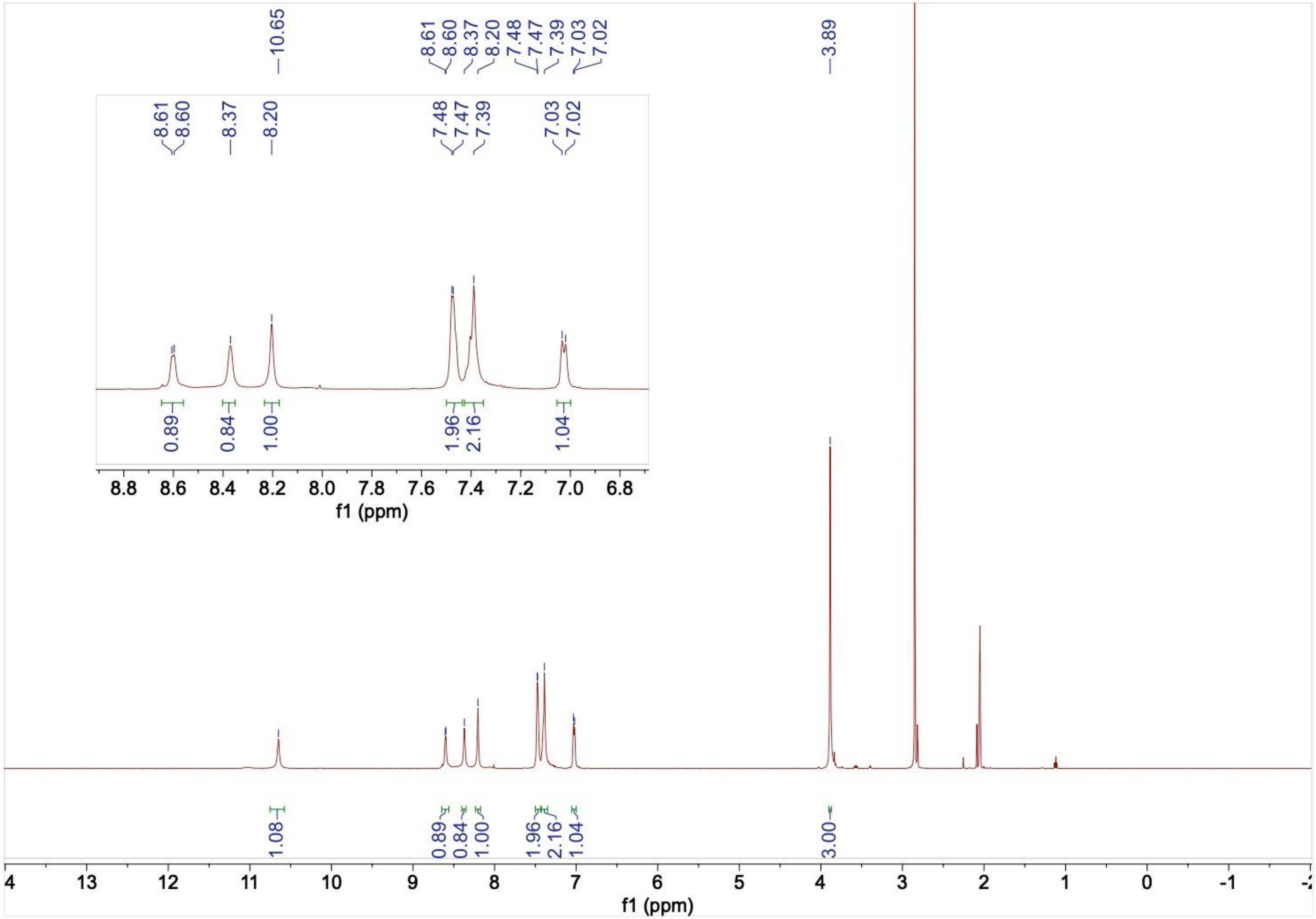

Figure S17. ^{1}H NMR spectrum (400 MHz, Acetone 25˚C): *N'*-(3-methoxybenzylidene)selenophene-2-carbohydrazide (**12**, LASSBio-2279).

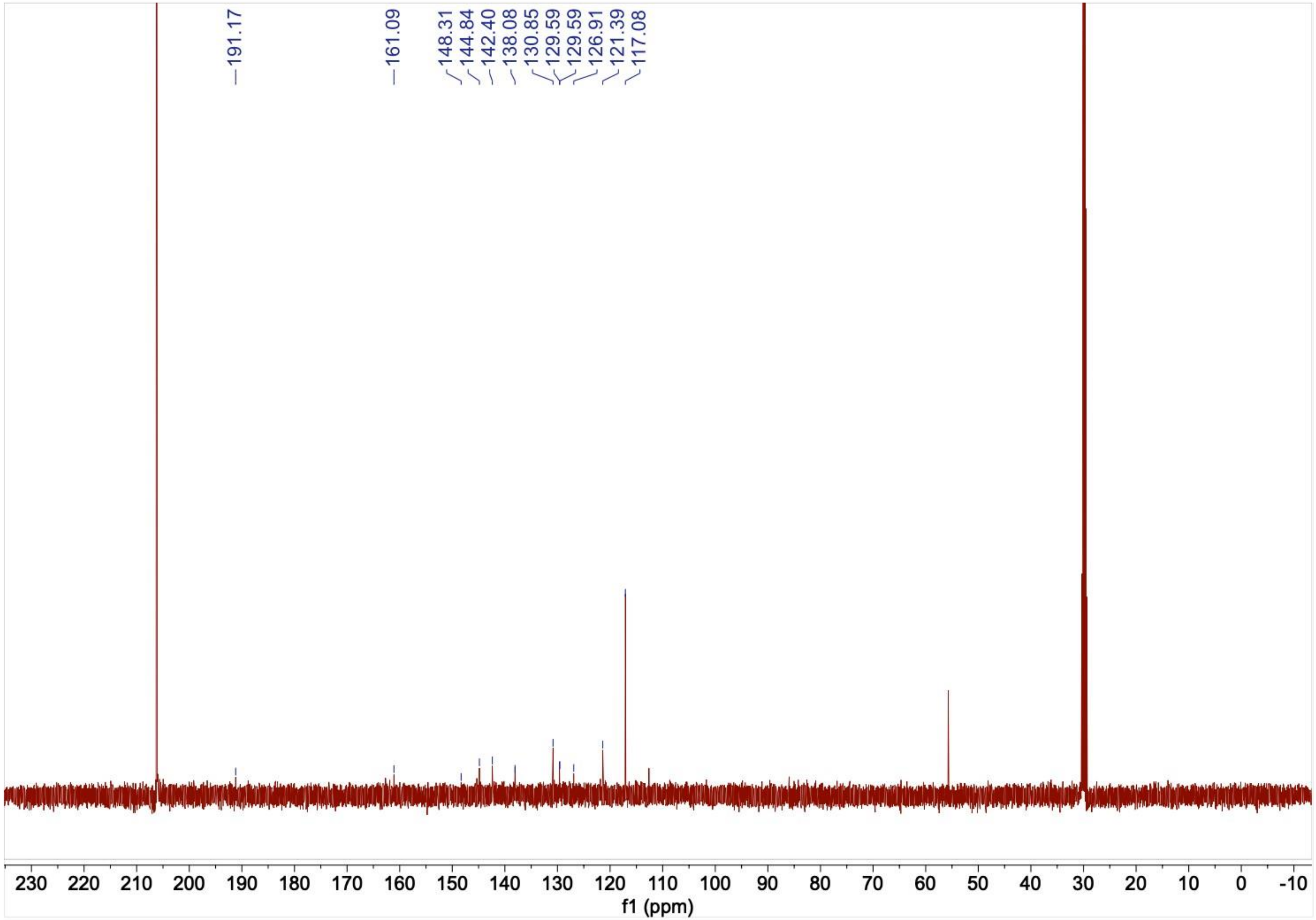

Figure S**18.** ^{13}C NMR spectrum (100 MHz, Acetone, 25˚C): *N*'-(3-methoxybenzylidene)selenophene-2-carbohydrazide (**12**, LASSBio-2279).

5. References

1. Yamada, S., Morizono, D., Yamamoto, K. Mild oxidation of aldehydes to the corresponding carboxylic acids and esters: alkaline iodine oxidation revisited *Tetrahedron Lett.* 1992;33:4329-4332.

2. Oertly, E., Ester, D. Über einige Derivate der Piperoysäure *Berichte der Deutschen Chemischen Gesellschaft*. 1910;43:1336-1340.

3. Hartman, R. J., Gassmann, A. G. Kinetics of the Estherification of Substituted Benzoic Acids *J. Am. Chem. Soc.* 1940;62:1559-1560.

4. Kümmerle, A. E., Síntese de compostos cardioativos 1,3-benzodioxolil-*N*-acilidrazônicos planejados por otimização do LASSBio-294, Federal University of Rio de Janeiro, Rio de Janeiro, 2005.

5. Hutton, K. The Synthesis of Some New Phenylurethans as Potential Local Anesthetics *J. Org. Chem.* 1955;7:855-861.

6. Omodei-Sale, A., Consonni, P., Galliani, G. A new class of nonhormonal pregnancy- terminating agents. Synthesis and contragestational activity of 3,5-diaryl-s-triazoles. *J. Med. Chem.* 1983;26:1187-1192.

7. Fischer, E., Speier, A. Darstellung der Ester *Berichte der Deutschen Chemischen Gesellschaft*. 1924;1924.

8. Andrisano, R., Pappalardo, G. Ultraviolet spectra of 2- and 3-monosubstituted derivatives of thiophenes *Bolletino Scientifico della Facolta di Chimica Industriale*. 1956;14:100-101.

9. Carrara, G., Chiancone, F. M., D'Amato, V., Ginoulhiac, E., Martinuzzi, C., Teotino, U. M., Visconti, N. Initial contribution to knowledge of the antitubercular activity of hydrazides *Gazzetta Chimica Italiana*. 1952;82:625-670.

10. Kornblum, N., Powers, J. W., Anderson, G. J., Jones, W. J., Larson, O. H. New and Selective Method of Oxidation *J. Am. Chem. Soc.* 1957;79:6562-6573.

11. Umezawa, S. Synthetic experiments in the selenophene groups. IV. Introduction of side chains into the selenophene nucleus *Bulletin of Chemical Society of Japan*. 1939;14:155-161.

12. Chierici, L., Pappalardo, G. Ultraviolet absorption spectra of alpha-monosubstitutedselenophenes *Gazzetta Chimica Italiana*. 1958;88:453-462.

13. Dubus, P., Decroix, B., Morel, J., Paulmier, C. Reactions of heterocyclic cyano derivatives *Comptes Rendus des Seances de l'Academie de Sciences, Serie C: Sciences Chimiques*. 1974;278:61-63.
